# Application of nanozyme-based drug delivery systems in the treatment of ROS-related ophthalmic diseases

**DOI:** 10.1016/j.ijpx.2026.100680

**Published:** 2026-10-08

**Authors:** Zhipeng Li, Keda Yan, Ning Rong, Shujie Sun, Xuemin Liu, Yu Xiu, Qiusheng Zheng, Yong Sun, Jingliang Wu

**Affiliations:** a School of Medicine, Weifang University of Science and Technology, Weifang 262700, China; b School of Life Science and Technology, Shandong Second Medical University, Weifang 261053, China; c Department of Pharmaceutics, School of Pharmacy, Qingdao University, Qingdao 266021, China; d Department of Dentistry, Shouguang Hospital of Traditional Chinese Medicine, Weifang 262700, China

**Keywords:** Nanozymes, Ophthalmic diseases, Drug delivery system, Oxidative stress, Responsive drug release

## Abstract

ROS-related ophthalmic diseases span the ocular surface as well as the anterior and posterior segments of the eye. Dry eye disease (DED), glaucoma, diabetic retinopathy (DR) and age-related macular degeneration (AMD) have distinct anatomical targets but share common pathophysiological features, including oxidative stress, inflammation, vascular dysfunction, epithelial injury or neurodegeneration. Conventional ocular drug delivery and therapeutic modalities are hindered by severe limitations, including low bioavailability in the posterior segment of the eye and the requirement for frequent invasive administration. Nanozymes, an emerging class of therapeutic agents that integrate the properties of nanomaterials with natural enzyme-like catalytic activity, can efficiently scavenge excess reactive oxygen species (ROS) in focal lesions, modulate inflammatory factors, and remodel the pathological microenvironment, thereby providing a novel approach to the treatment of ROS-related ophthalmic diseases. This review systematically elucidates the core characteristics and therapeutic mechanisms of four major categories of nanozymes, namely metal-based, metal oxide-based, carbon-based, and composite nanozymes. To overcome physiological barriers in ocular drug delivery, such as the cornea and the blood-retinal barrier (BRB), we discuss active targeted delivery strategies based on mucin-binding retention, biomimetic cell membrane coating, and molecular ligand-mediated targeting. Furthermore, we summarize the development of intelligent and controlled-release systems in response to specific pathological microenvironments, such as ROS, pH values, and others. Additionally, this review details the latest cutting-edge applications and therapeutic efficacies of nanozymes in specific ophthalmic diseases, including AMD, DR, dry eye disease, and glaucoma. Finally, we highlight the challenges of long-term biosafety evaluation in the bench-to-bedside clinical translation of nanozymes and provide perspectives on future research directions for developing multi-stimuli-responsive synergistic nanodelivery systems.

## Graphical abstract

**Figure:**
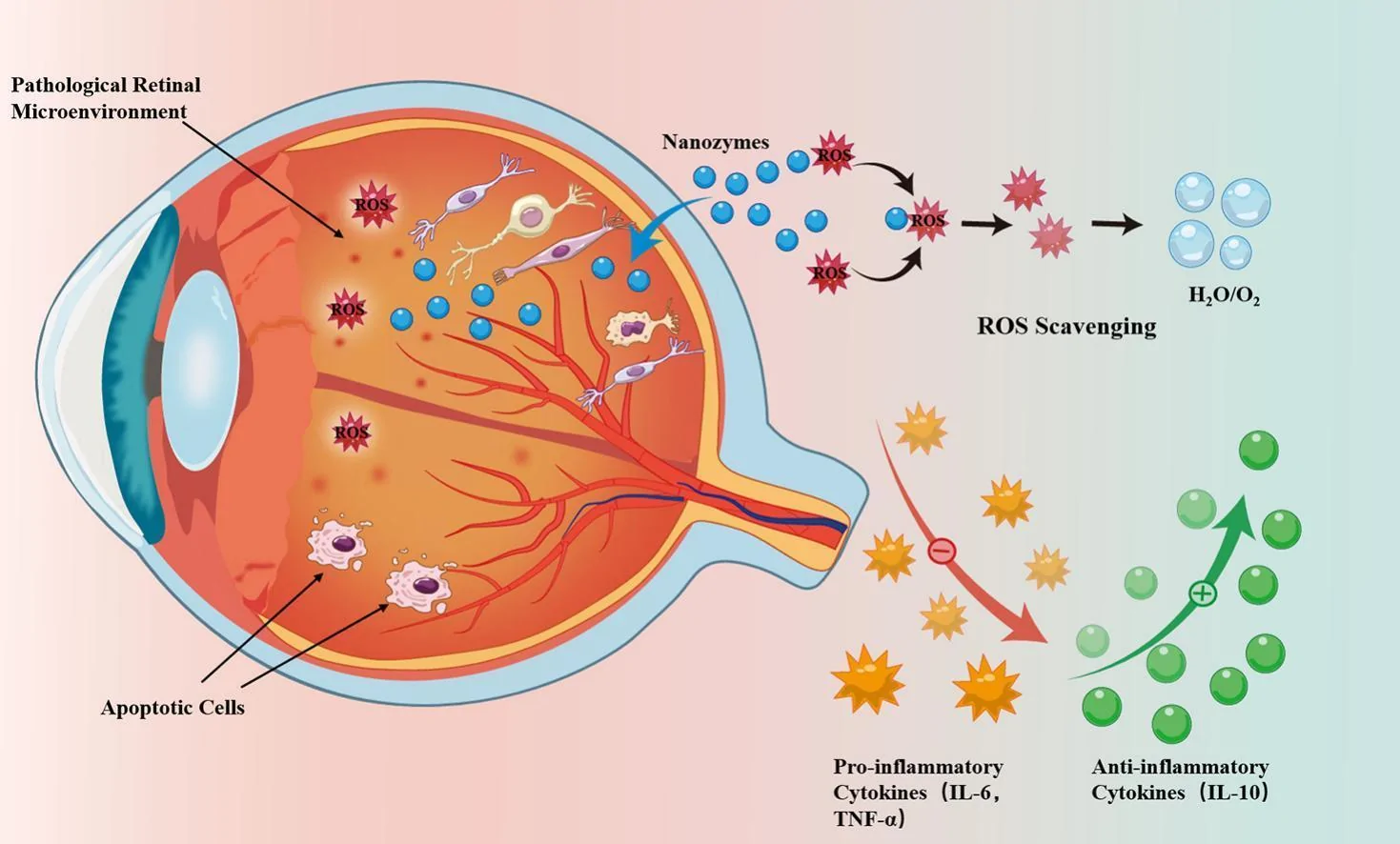

## 1 Introduction

Ophthalmic diseases have emerged as a major global public health concern, with their incidence exhibiting a steady upward trajectory (Hendrick et al., 2015; Infeld and O'Shea, 1998). According to data from the World Health Organization (WHO), the global population afflicted by retinal diseases has surpassed 300 million, profoundly impacting patients' quality of life. Representative ROS-related diseases discussed in this review include diabetic retinopathy, age-related macular degeneration, dry eye disease, glaucoma and retinal ischemia-reperfusion (I/R) injury. These diseases affect different ocular compartments but converge on redox imbalance, inflammation, epithelial, vascular or neurodegenerative injury (Jiang et al., 2025; Shukla and Tripathy, 2026; Xu et al., 2025). Among them, the pathogenesis of diabetic retinopathy is a highly complex and multifactorial process, involving metabolic disorders, oxidative stress, inflammatory responses, vascular dysfunction, and neurodegeneration (Kang and Yang, 2020; Kour et al., 2024; Liu et al., 2022a; Yu, 2026).

The retina possesses a complex and highly stratified cellular architecture, comprising photoreceptors (rods and cones), horizontal cells, bipolar cells, amacrine cells, and retinal ganglion cells (Fig. 1A). Under pathological stress conditions, such as hyperglycemia or hypoxia, resident ramified microglia within the retina are rapidly activated and polarize toward a pro-inflammatory M1 phenotype (Li et al., 2025a; Sun et al., 2025). This activation triggers the massive release of ROS and pro-inflammatory mediators, including TNF-α, IL-1β, COX-2, and iNOS (Fig. 1B) (Kakkar et al., 2025). Concurrently, this pathological cascade suppresses the anti-inflammatory and tissue-reparative M2 phenotype (characterized by the expression of markers such as CD206, IL-10, and PPAR). The consequent immunological imbalance, coupled with sustained oxidative stress, severely exacerbates the deterioration of the retinal microenvironment (Ma and Shen, 2025; Zhang et al., 2025a).

**Fig. 1:**
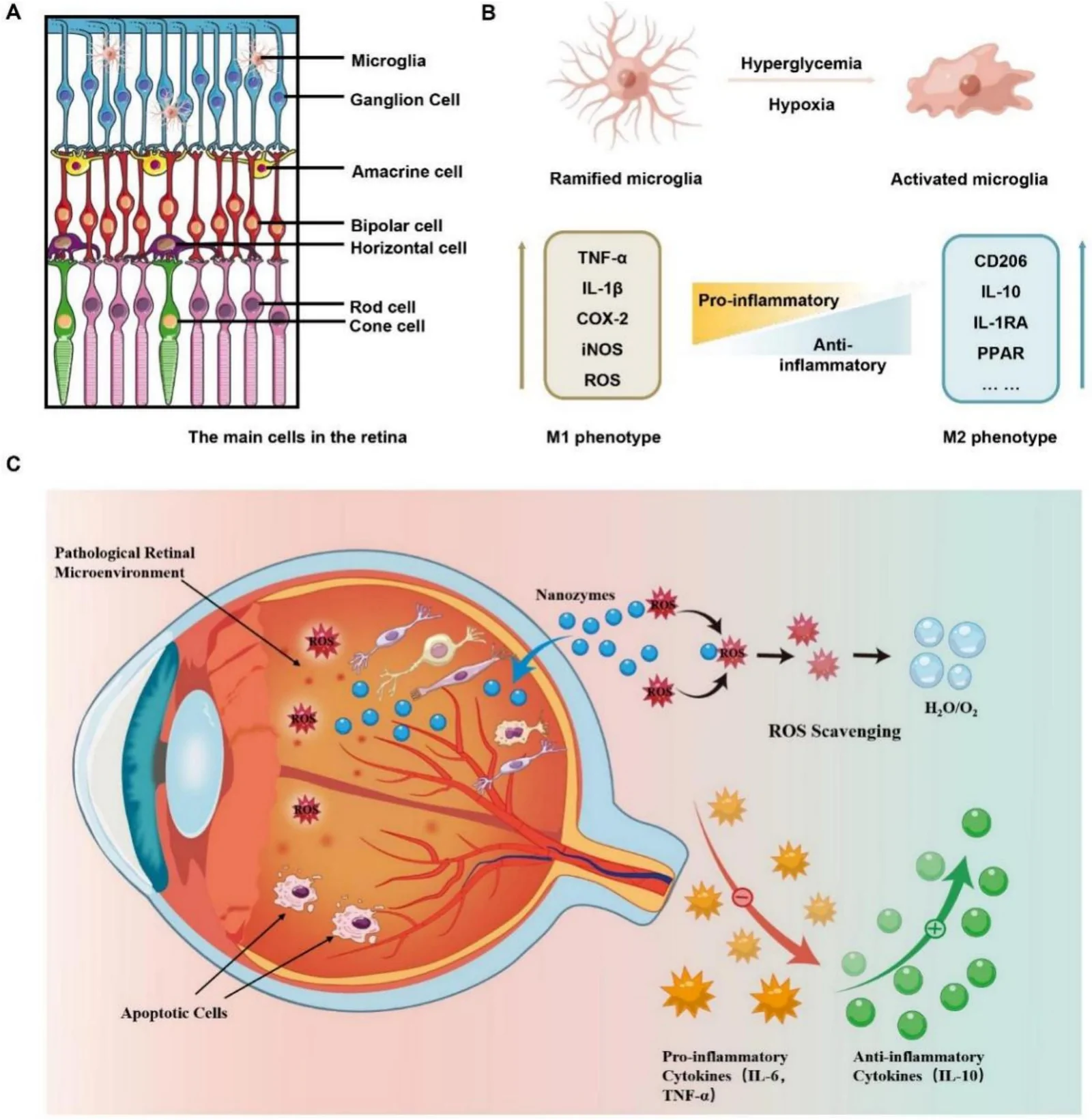
(A) The main cell types in the retina; (B) Under pathological conditions, microglia become activated and polarize into pro-inflammatory M1 phenotype, releasing corresponding inflammatory factors; (C) Nanomachines eliminate ROS, alleviate retinal inflammation, inhibit the formation of new blood vessels, and reduce cell apoptosis.

Therapeutic strategies differ across ophthalmic diseases. For neovascular retinal diseases such as neovascular AMD or diabetic macular edema, although repeated injections and incomplete or variable responses remain important therapeutic burdens, intravitreal VEGF inhibition remains the clinical standard and provides established visual and anatomical benefits. In contrast, DED and glaucoma require disease-specific approaches directed toward ocular surface homeostasis, inflammation, aqueous humor outflow or neuroprotection. These limitations across distinct ocular compartments motivate complementary delivery and disease-modifying strategies rather than a single replacement therapy.

As an emerging class of nanoscale therapeutics, nanozymes exhibit diverse enzyme-mimicking activities, including peroxidase (POD), catalase (CAT), superoxide dismutase (SOD), and oxidase (OXD) (Gu et al., 2025; Thakur et al., 2026). By mimicking the active sites of natural enzymes, they leverage the inherent surface properties of nanomaterials to catalyze specific chemical reactions. Under mild physiological conditions, nanozymes can efficiently catalyze corresponding substrates to yield products identical to those of their natural counterparts, showing tremendous utility in disease diagnosis and therapy. Compared to natural enzymes, nanozymes possess superior attributes, such as robust stability, facile synthesis, excellent recyclability, and ease of surface modification, which substantially lower the technical barriers and financial burdens associated with clinical translation (Li et al., 2025b). More importantly, precise surface engineering endows nanozymes with capabilities for targeted delivery and functional regulation, thereby unlocking their potential for treating a wide array of diseases (Feng et al., 2025). In 2018, Das et al. constructed functionally differentiated nanozymes using gold nanoparticles as scaffolds via precise surface modification. Specifically, by employing cationic and zwitterionic modifications to modulate cellular uptake efficiencies, they achieved intracellular ROS scavenging and extracellular inflammatory microenvironment modulation (Das et al., 2019).

It should be emphasized that different oxidoreductase-like activities of nanozymes have fundamentally different consequences for redox homeostasis. SOD-like activity catalyzes the dismutation of O_2_^•-^ into H_2_O_2_ and O_2_, whereas CAT-like activity subsequently decomposes H_2_O_2_ into H_2_O and O_2_. Therefore, coupling these two activities can establish a sequential antioxidant pathway to remove major ROS species. In contrast, POD-like and OXD-like activities may exert pro-oxidant effects under specific conditions. Many POD-like nanozymes catalyze H_2_O_2_-dependent oxidation, particularly in Fenton- or Fenton-like systems, which generate highly reactive •OH, while OXD-like nanozymes can reduce molecular oxygen to ROS such as O_2_^•-^ or H_2_O_2_. Such ROS-generating activities are advantageous for applications requiring oxidative damage including antibacterial or anti-tumor therapy, but it may be undesirable in retinal and ocular surface diseases where excessive oxidative stress is already a major pathological driver (Stasyuk et al., 2022). Accordingly, antioxidant ophthalmic nanozymes should not simply maximize total catalytic activity, but should prioritize enhancing SOD- and CAT-like pathways while minimizing net ROS generation through POD- and OXD-like pathways under ocular physiological and pathological conditions. This selectivity can be achieved by regulating the composition and valence state of catalytic center, engineering surface ligands or polymer coatings, controlling exposed crystal facets and active-site accessibility, and exploiting the pH dependence of competing catalytic pathways. Therefore, catalytic selectivity, rather than nominal multi-enzyme activity, should be considered a key design criterion for ophthalmic antioxidant nanozymes.

The unique physiological barrier system of the eye represents a core challenge for drug delivery to the retina. The tight junctions of the corneal epithelium and the highly selective permeability of the BRB result in a fundamental bioavailability of less than 5% for conventional administration routes. The emergence of nanozymes offers an innovative solution to this challenge (Zhao et al., 2026). The FR-PolyRu nanozyme eye drops developed by Xue et al., through fluorination modification to reduce transmembrane activation energy and RGD peptide-mediated targeting of vascular endothelial cells, combined with the transport driving force provided by catalytic oxygen generation, successfully achieved efficient penetration of the nanozyme across the corneal and BRB barriers following non-invasive topical administration. In a mouse model of oxygen-induced retinopathy, this strategy reduced the non-perfusion area by more than 40%. This delivery strategy fundamentally overcomes the limitations of invasive conventional anti-VEGF therapy and greatly improves patient compliance (Xue et al., 2025).

At the level of therapeutic mechanisms, nanozymes exhibit multiple advantages characterized by catalytic regulation and microenvironment remodeling (Fig. 1C). Upon delivery into the pathological retinal microenvironment characterized by abundant ROS and extensive apoptotic cells, nanozymes exert potent targeted scavenging capacity, efficiently catalyzing the conversion of excessive toxic ROS into harmless H_2_O and O_2_(Su et al., 2026; Wang et al., 2025; Yang et al., 2025). This in situ catalytic action not only directly rescues retinal cells from apoptosis but also drives the reversal of the local immune microenvironment by significantly suppressing the expression of pro-inflammatory cytokines (such as interleukin-6 and tumor necrosis factor-α) while concurrently upregulating anti-inflammatory cytokines (IL-10) (Zhou et al., 2026).The iron-quercetin nanozyme developed by Gui et al., which possesses SOD, CAT and POD-mimicking triple enzymatic activities, reduced retinal ROS levels by 40% in a diabetic rat model while downregulating the expression of inflammatory factors such as IL-6 and TNF-α, thereby inhibiting vascular endothelial growth factor (VEGF)-mediated vascular leakage (Gui et al., 2023).

Although nanozymes have achieved preliminary progress in the treatment of retinopathies, their clinical translation still faces challenges including biosafety assessment, validation of long-term in vivo stability, and optimization of personalized administration regimens (Wu et al., 2019; Zhang et al., 2019). With continuous innovations in material synthesis technologies and targeted delivery systems, nanozymes are expected to achieve the transition from laboratory research to clinical applicability, offering safer and more effective therapeutic options for hundreds of millions of patients with retinopathies worldwide. This review systematically summarizes nanozyme-based delivery and therapy across ROS-related ophthalmic diseases affecting the ocular surface, anterior segment and posterior segment, with representative applications in DED, glaucoma, DR, AMD and retinal vascular injury. It begins by describing the properties and advantages of various nanozymes, with a focus on their catalytic mechanisms and delivery strategies, and finally discusses their applications in specific diseases, aiming to provide a reference for future research in this field.

## 2 The types and characteristics of nanozymes

Nanozymes are a type of nanomaterial that possesses the unique physical properties of nanomaterials and also exhibits enzymatic catalytic activity (Wang et al., 2019). Based on their core components and structural characteristics, nanozymes can be classified into four major categories: metal-based nanozymes, metal oxide nanozymes, carbon-based nanozymes, and composite nanozymes (Table 1).

**Table 1:** Summary of structural characteristics, preparation and applications of different types of nanozymes.

Category	Representative materials	Main property	Preparation method	Catalytic mechanism	Key quantitative parameters
Metal-based nanozymes	Au, Pt, Pd, Ag, Fe, Co, Cu	1. Good stability and stable chemical properties; 2. High-density active sites, high catalytic efficiency and strong reaction selectivity; 3. Possesses surface plasmon resonance characteristics and excellent optical and photothermal conversion properties; 4. The preparation cost of metal alloy nanozymes is low and the synthesis process is simple.	High-temperature reduction method, Electrochemical synthesis, Photochemical method, Biological synthesis, Co-precipitation method, Sol-gel method, Thermal decomposition method.	By utilizing the coordination effect and electron transfer ability of atoms on the metal surface, the enzymatic activities of SOD, CAT, POD, etc. can be simulated.	1. PtNPs-BSA: ∼20 nm; POD/CAT/SOD-like activities. 2. Fe-SANzyme: ∼50 nm; CAT/SOD-like activities. 3. In Cyano@Au@Ir, Au and Ir nanoparticles were approximately 15 nm and 1.5 nm respectively, supporting a spatially coupled GOx-like/CAT-like cascade.
Metal oxide nanozymes	CeO_2_, Fe_3_O_4_, MnO_2_	1. The active core is the variable valence state of metal ions, which can achieve continuous catalysis through valence state cycling; 2. It has high chemical stability and durability, and maintains catalytic activity even after long-term storage; 3. The raw material cost is low, making it suitable for large-scale production and clinical transformation.	Co-precipitation method, Sol-gel method, Thermal decomposition method.	By simulating enzyme activity through the cycling of metal ion valence states, ROS are eliminated and oxidative stress is regulated.	1. Cu_5.4_O: 4.47 ± 1.19 nm; SOD-/CAT-like activities; ∼80% H_2_O_2_, ∼30% O_2_^•-^ and ∼ 20% •OH scavenging at 1 μg/mL. 2. Fe-Quer: ∼5–10 nm; SOD-/CAT-/POD-like activities; >90% DPPH/ABTS radical scavenging. 3. G/Ce-XL: Ce core 73.5 ± 6.3 nm; CAT- and SOD-like activities.
Carbon-based nanozymes	Single-atom carbon, Surface-functionalized carbon, Doped carbon, Composite carbon-based materials	1. High chemical stability, maintaining catalytic activity even under high temperatures and extreme pH conditions; 2. Wide range of raw material sources with low cost; 3. Good biocompatibility and low toxicity; 4. Rich in carboxyl and hydroxyl groups on the surface, which can be modified to achieve multifunctionality.	Doping with non-metallic elements, Surface modification, Combination with other materials.	The synergistic effect of the delocalized electron system based on the carbon skeleton and the surface functional groups enables the simulation of enzyme activities such as POD and OXD through electron transfer. POD/OXD-like activity: potentially ROS-generating, depending on substrate and reaction pathway. SOD/CAT-like activity: predominantly ROS-scavenging.	1. C-dots@Gel: ∼3–5 nm; SOD-like activity; inhibition ∼90% at 40 μg/mL; ABTS scavenging >60% at 150 μg/mL. 2. TM-CDs: 1.9 nm (TEM), 7.5 nm (DLS); CAT- and SOD-like activities.
Composite nanozymes	Metal-organic frameworks with peptide conjugation or antibody conjugation	1. Integrate multiple enzyme activities to achieve cascade reactions; 2. Enhance targeting through the coupling of biological molecules; 3. Can be combined with multimodal treatments such as photothermal and sonodynamic therapies.	Coupling method, Self-assembly method, Composite synthesis method.	The synergistic action of multiple enzyme activities enables precise treatment through cascade reactions or multiple stimulus-response mechanisms.	1. Pt-MOF/Au@HA: 256 ± 35 nm; GOx-/CAT-/SOD-like activities; O_2_ generation ∼3.3 mg/L and glucose decrease ∼4.5 mmol/L. 2. Pt-Pd-Au: 44.5 ± 5.6 nm (DLS); OXD- and POD-like activities.

### 2.1 Metal-based nanozymes

Metal-based nanozymes are represented by noble metal nanoparticles such as gold (Au), platinum (Pt), palladium (Pd), and silver (Ag), and some transition metal nanoparticles (such as Fe, Co, Cu) also fall into this category. Metal-based nanozymes can be classified as single-metal nanozymes and metal alloy nanozymes. Single-metal nanozymes exhibit good stability under natural conditions and possess significant chemical stability. The high density of active sites and the uniform catalytic environment significantly enhance the catalytic efficiency and reaction selectivity. Moreover, single-metal nanozymes also have remarkable surface plasmon resonance characteristics, excellent optical and photothermal conversion properties, thus enabling optical treatment of diseases (Chen et al., 2019a; Liu et al., 2021b). Single-metal nanozymes can be prepared through various methods such as high-temperature reduction, electrochemical synthesis, photochemical synthesis, and biological synthesis. On the other hand, metal alloy nanozymes have a relatively lower preparation cost and a simpler synthesis process, and their catalytic performance is more outstanding than that of single-metal nanozymes. Their preparation methods are diverse, including co-precipitation method, sol-gel method, and thermal decomposition method, etc.

The catalytic activity of metal-based nanozymes stems from the coordination effect and electron transfer ability of the atoms on the metal surface. They exert catalytic effects by mimicking the enzymatic activities (such as SOD, CAT, POD), and the activity of nanozymes is not only related to the inherent properties of the material, such as the constituent elements, size, and morphology, but also influenced by external factors (pH value, temperature, light) (Zeng et al., 2024). Tarricone et al. developed a complex formed by combining platinum nanoparticles with bovine serum albumin, which not only significantly enhanced their stability in physiological environments but also improved their uptake by neuronal cells. Notably, under simulated lysosomal acidic conditions, the catalase-like activity of the platinum nanozyme substantially exceeded that of cerium oxide nanoparticles under equivalent conditions. Furthermore, in vitro experiments demonstrated that platinum nanozymes coated with bovine serum albumin effectively reduced intracellular reactive oxygen species accumulation induced by hydrogen peroxide (H_2_O_2_) or antimycin A in neurons and suppressed associated apoptotic signaling pathways, exhibiting potent antioxidative stress protective effects (Tarricone et al., 2023).

To overcome the challenges of easy aggregation of single atoms and the low utilization efficiency caused by active sites being buried within dense carbon matrices, Zhang et al. proposed an “edge-site engineering” strategy (Zhang et al., 2022). Using a pyrolysis-leaching method, they prepared a hierarchically mesoporous Fe-SANzyme enriched with defective Fe—N_4_ sites (Fig. 2). In a mouse model of retinal vascular disease, following intravitreal injection, the Fe-SANzyme reached the lesion site and efficiently decomposed pathologically accumulated H_2_O_2_ into O_2_ and H_2_O through its catalase-like activity. This not only effectively scavenged ROS to alleviate oxidative stress but also achieved functional normalization of pathological vasculature by inhibiting the aberrant expression of vascular endothelial growth factor, without exerting significant effects on normal retinal blood vessels. Besides, Zhou et al. developed a cyanobacteria-loaded Au NPs composite system (Cyano@Au@Ir NPs) that demonstrated multidimensional regulatory advantages. The Au NPs first degraded glucose into hydrogen peroxide, which was subsequently decomposed into H_2_O and O_2_ by Ir NPs, constituting a cascade hypoglycemic reaction. The cyanobacteria continuously produced O_2_, forming a synergistic closed loop of “glucose reduction-toxin elimination-oxygen supply”. Moreover, this system significantly reduced pathological angiogenesis and downregulated the aberrant expression of hypoxia-inducible factor-1α (HIF-1α) and VEGF, thereby inhibiting pathological neovascularization (Zhou et al., 2023).

**Fig. 2:**
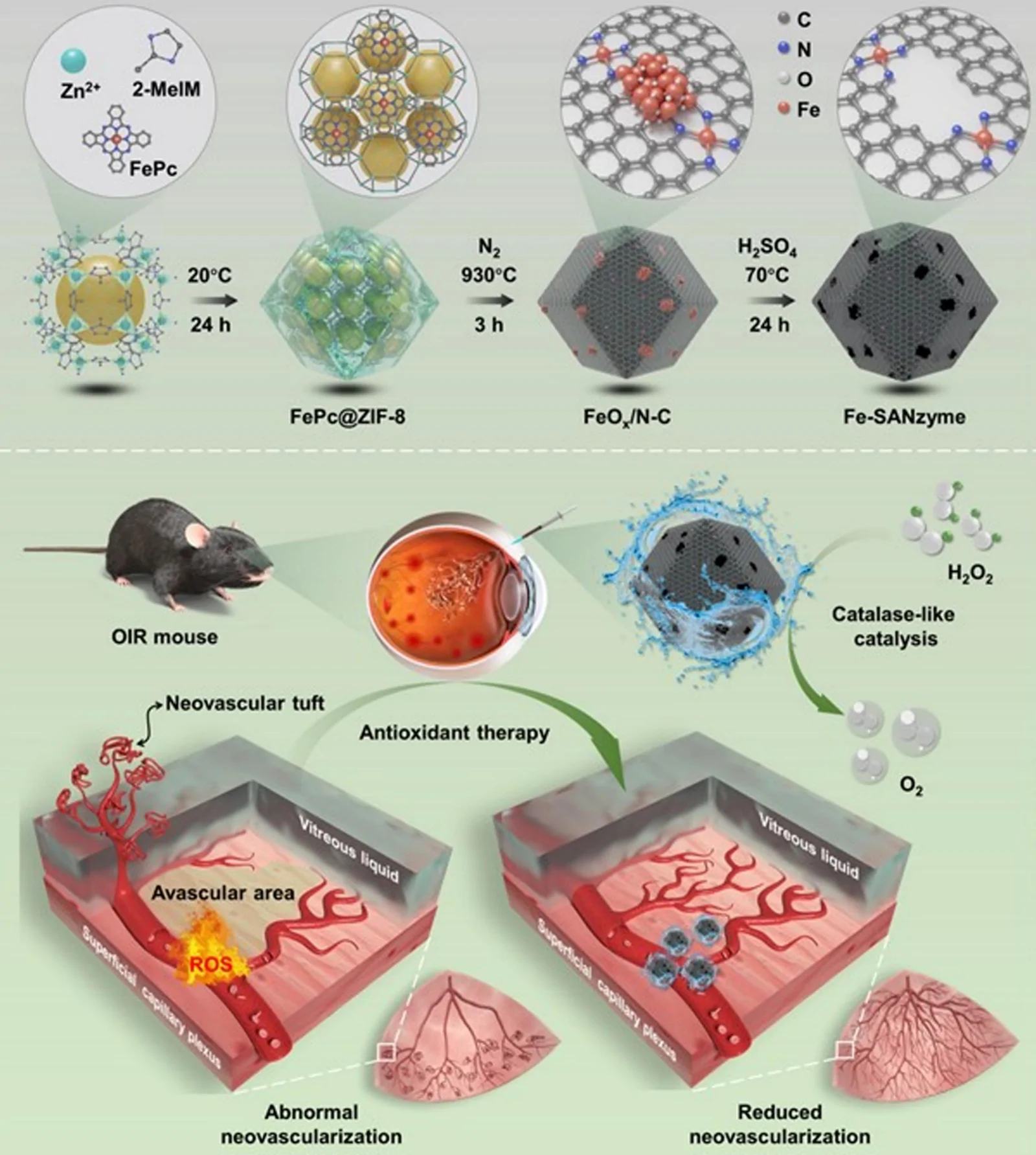
Schematic illustration of the synthesis process and therapeutic effects of Fe-SANzyme. This scheme links active-site engineering with ROS removal and normalization of pathological retinal vessels. This figure has been reproduced from ref. Zhang et al. (2022) with permission from Wiley, copyright 2022.

### 2.2 Metal oxide nanozymes

Metal oxide nanozymes include cerium oxide (CeO_2_), iron(III) oxide (Fe_3_O_4_), manganese oxide (MnO_2_), etc.(Chen et al., 2019b; Liu et al., 2021a). The active core of these nanozymes is the variable valence state of metal ions, which can achieve continuous catalytic reactions through valence state cycling. Compared with single-metal nanozymes, metal oxide nanozymes have higher chemical stability and durability. They can maintain their catalytic activity even under long-term storage conditions, and the cost of raw materials required for their preparation is lower, which is a crucial factor for large-scale production and eventual clinical translation (Mathur et al., 2024). Qiao et al. prepared an ultrasmall Cu_5.4_O nanozyme eye drop with a particle size of approximately 4.47 nm, which effectively scavenged ROS on the ocular surface, significantly downregulated the expression of pro-inflammatory cytokines such as TNF-α and IL-1β, and upregulated the level of the anti-inflammatory cytokine IL-10. This formulation reduced inflammatory cell infiltration in corneal tissue, restored corneal epithelial thickness and cell density, and ameliorated the disorganized arrangement of stromal collagen fibers, thereby achieving protection against corneal oxidative injury and promoting epithelial regeneration (Qiao et al., 2025).

The redox cycling of multivalent metal centers should be interpreted as a conditional catalytic process rather than as unlimited self-regeneration. For nanoceria, SOD- and CAT-like activities arise from reversible surface Ce^3+^/Ce^4+^ conversion coupled to oxygen vacancy chemistry. Among them, the relative abundance and accessibility of Ce^3+^ and Ce^4+^ sites play a dominant role in influencing catalytic activity. This cycle can be repeatedly restored when surface cerium remains redox, but it is sensitive to the surrounding chemical environment. H_2_O_2_ exposure can transiently shift surface cerium toward Ce^4+^, whereas phosphate can bind Ce^3+^ sites and suppress Ce^3+^/Ce^4+^ interconversion. In addition, pH value, adsorbed biomolecules or surface aging can also alter activity. Thus, long-term catalytic persistence reflects reversible surface redox chemistry and preservation of accessible oxygen-vacancy sites rather than an inexhaustible catalytic capacity (Nelson et al., 2016). Similar restrictions also apply to Fe^2+^/Fe^3+^ cycle in iron-based nanozymes. Mixed-valence iron centers can support electron transfer and fenton/peroxidase-like reactions, but regeneration of Fe^2+^ depends on the local redox environment and available electron donors, while oxidation, leaching, aggregation or surface passivation may progressively change catalytic flux (Gao et al., 2007). Gui et al. developed an ultrasmall coordination polymer nanodot nanozyme named iron-quercetin (Fe-Quer), which mimics superoxide dismutase, catalase, and peroxidase activities. This design ingeniously combines the intrinsic pharmacological activity of quercetin with the catalytic activity of iron ions. Experimental results demonstrated that this nature-inspired biomimetic strategy effectively intervenes in key pathological events in the progression of diabetic retinopathy, including oxidative stress, inflammation, and pathological angiogenesis (Gui et al., 2023). Although Fe-Quer exhibited measurable SOD-, CAT- and POD-like activities, its therapeutic outcomes in diabetic retinopathy were characterized by a reduction in oxidative stress. This example illustrates that the biological consequences of multi-enzyme nanozymes depend on the relative catalytic fluxes of competing antioxidant and pro-oxidative pathways, rather than merely on the presence of specific enzyme-like activity. Therefore, future ophthalmic studies should quantify catalytic selectivity and ROS production of multi-enzyme nanozymes under disease-relevant pH and substrate conditions when evaluating them.

Yang et al. developed a gabapentin-loaded cerium oxide nanocomposite (G/Ce-XL) that utilizes the catalase-like and superoxide dismutase-like activities of CeO_2_ to efficiently scavenge ROS and modulate oxidative stress (Fig. 3). The study confirmed that thiolated gelatin-modified cerium oxide nanoparticles significantly inhibited H_2_O_2_-induced intracellular reactive oxygen species production, reduced pro-inflammatory cytokine levels, and suppressed pathological neovascularization (Yang et al., 2023).

**Fig. 3:**
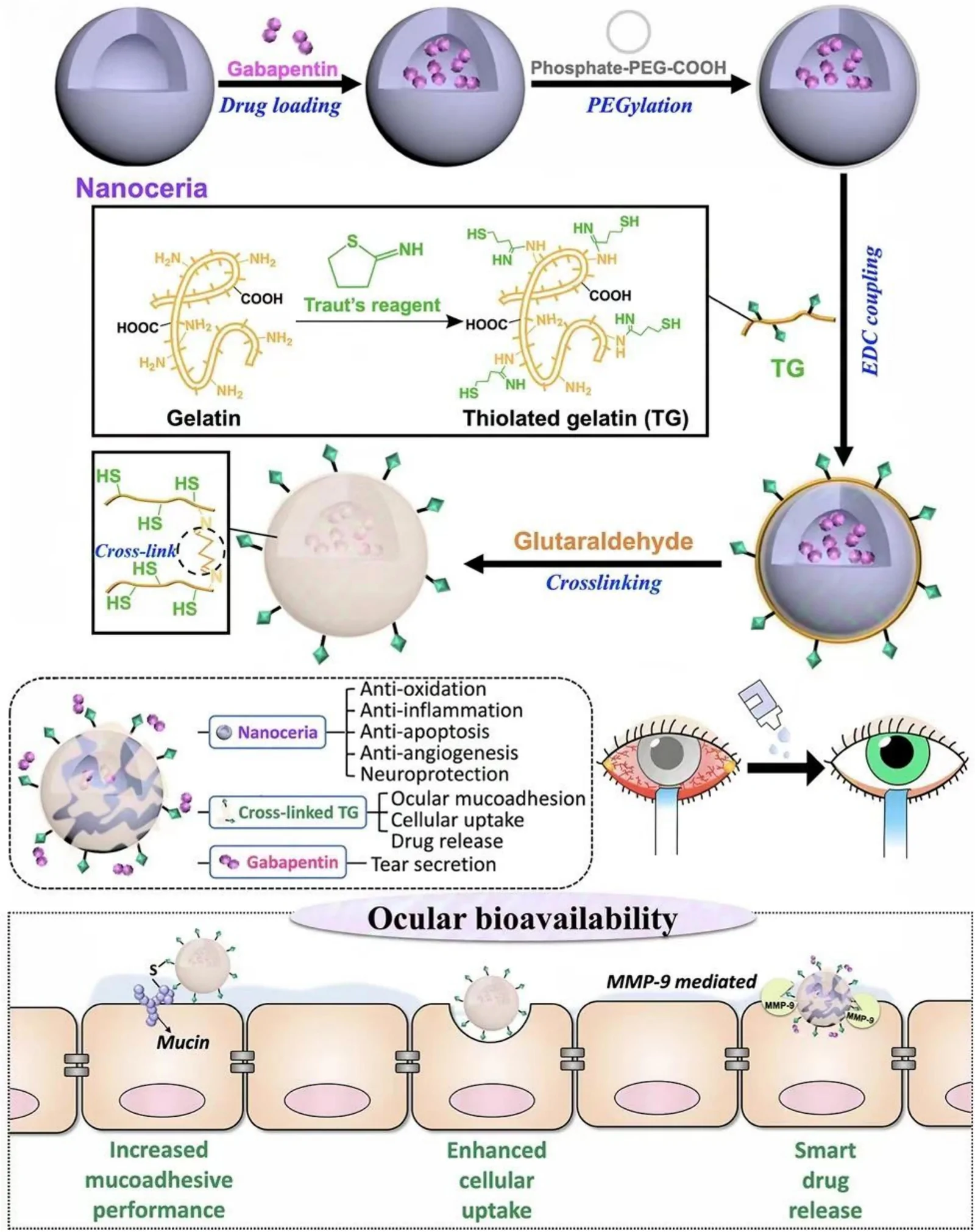
Preparation and ocular action of G/Ce-XL. This schematic diagram highlights the integration of nanoceria-mediated ROS scavenging, thiolated-gelatin mucoadhesion and controlled gabapentin delivery for dry eye therapy. This figure has been reproduced from ref. Yang et al. (2023) with permission from ACS, copyright 2023.

### 2.3 Carbon-based nanozymes

Carbon-based nanozymes generally possess higher chemical stability and maintain robust catalytic activity even under harsh conditions such as high temperatures and extreme pH levels (Ding et al., 2020; Sun et al., 2022b; Zhuang et al., 2024). Their raw materials are widely available and cost-effective, with a cost significantly lower than that of noble metals such as Au and Pt. Not only that, carbon-based nanozymes exhibit excellent biocompatibility and low biotoxicity. Owing to their abundant carboxyl and hydroxyl groups, heteroatom doping and surface modification of the carbon skeleton allow for effective modulation of their catalytic activity and selectivity (Feng et al., 2024; Ye et al., 2025). Carbon-based nanozymes can not only mimic a single enzyme activity but also integrate multiple enzymatic activities, including peroxidase-like, oxidase-like, catalase-like, and glucose oxidase-like properties. They mainly include single-atom carbon nanozymes, surface-functionalized carbon nanozymes, doped carbon nanozymes, and composite carbon-based nanozymes, with the possibility of incorporating other metallic or non-metallic materials to synergistically enhance their catalytic performance (Fu et al., 2024; Wang et al., 2021). Based on the presence or absence of metal elements, they can be categorized into metal-free and metal-based carbon-based nanozymes. Metal-free carbon-based nanozymes primarily rely on the synergistic interplay between the delocalized electron system of the carbon skeleton and surface functional groups to drive catalytic processes, mimicking peroxidase-like or oxidase-like activities through electron transfer mechanisms (Li et al., 2023; Sun et al., 2022a; Zhou et al., 2025).

Ocular specific studies have begun to demonstrate the therapeutic relevance of carbon-based nanozymes. Wei et al. incorporated superoxide dismutase-like carbon dots into a thermosensitive in situ hydrogel (C-dots@Gel) for dry eye disease. The hydrogel increased adhesion and residence on the corneal surface, while carbon-dot nanozyme reduced oxidative stress, improved tear-film stability, tear secretion, corneal epithelial integrity and conjunctival goblet cell density in a benzalkonium chloride-induced mouse model (Wei et al., 2024). More recently, Zhu et al. prepared amphiphilic carbon dots derived from cherry tomatoes (TM-CDs) with multi-enzyme-like antioxidant activity. These carbon dots accumulated in ocular tissues of zebrafish, which scavenged multiple ROS species, increased SOD activity and glutathione levels, reduced malondialdehyde accumulation, and ameliorated oxidative ocular injury (Zhu et al., 2025a). Together, these studies provide direct evidence for the ocular applications of carbon-based nanozymes. Although the existing data are still mainly preclinical, further validation is needed in mammalian retinal and ocular surface models.

### 2.4 Composite nanozymes

By conjugating two or more nanomaterials or biomolecules (such as antibodies or peptides) with nanozymes, enhanced catalytic activity or functional targeting can be achieved (Gopakumar et al., 2025; Li et al., 2021; Maddheshiya and Nara, 2022). Composite nanozymes integrate multiple enzymatic activities to enable cascade reactions. Tian et al. designed a Pt-MOF/Au@HA composite nanozyme system based on metal-organic framework materials, integrating the glucose oxidase-like activity of gold nanoparticles with the catalase-like and superoxide dismutase-like activities of platinum nanoparticles within a single system. This significantly enhanced its therapeutic potential for diseases associated with metabolic abnormalities, enabling efficient glucose decomposition, robust scavenging of excessive ROS, and synergistic coordination of hypoxia alleviation and anti-inflammatory effects in the lesion area, demonstrating substantial advantages in the treatment of diseases characterized by microenvironmental imbalance, such as diabetic retinopathy (Tian et al., 2025).

He et al. developed a Pt-Pd-Au trimetallic alloy composite nanozyme (PPA) that exerts multimodal synergistic antibacterial effects through photothermal therapy, chemodynamic therapy, and sonodynamic therapy. The near-infrared-induced high photothermal conversion efficiency of 52.21% enhanced catalytic activity and promoted deep tissue penetration, while the cavitation effect of ultrasound reduced mass transfer resistance and further amplified enzymatic activity. The synergistic action of these modalities achieved a bacterial inhibition rate of 95%. The antibacterial mechanism involves regulation of oxidative stress pathways, interference with quorum sensing systems, and reprogramming of metabolic networks. Concurrently, this composite nanozyme promoted macrophage M1-to-M2 polarization and angiogenesis, accelerating wound healing in deep-seated drug-resistant bacterial infections while maintaining excellent biocompatibility (He et al., 2025).

## 3 Ocular targeting strategies for nanozymes

Although nanozymes exhibit considerable potential in mitigating oxidative stress and inflammation for the treatment of ophthalmic diseases, their non-specific in vivo distribution can lead to off-target effects and impede their ability to traverse the complex physiological barriers of the eye, such as the blood-retinal barrier, thereby limiting their therapeutic efficacy (Xue et al., 2022; Xue et al., 2024). Unmodified nanozymes administered via eye drops struggle to penetrate tight junction structures, while systemic administration renders them susceptible to clearance by the reticuloendothelial system (Rong et al., 2018; Wu et al., 2026). This results in insufficient drug accumulation at the retinal lesion site and concurrently elevates the risk of peripheral tissue toxicity (Okur et al., 2025).

Beyond the cornea and BRB, ocular delivery is constrained by a sequence of dynamic and anatomical barriers. Following topical instillation, formulations are rapidly diluted by basal and reflex tears, redistributed by blinking and removed through nasolacrimal drainage. The mucin rich glycocalyx can retain nanoparticles or hinder their epithelial penetration depending on particle size, surface charge and surface chemistry. In addition to the transcorneal route, topically administered nanozymes may access intraocular tissues via conjunctival-scleral pathway, but conjunctival vascular and lymphatic drainage can divert part of the dose into the systemic circulation. Inside the eye, aqueous humor turnover, blood-aqueous barrier, vitreous matrix, inner limiting membrane, retinal extracellular matrix, BRB and choroidal circulation further shape distribution, retention and clearance. Thus, enhanced corneal permeability alone does not necessarily translate into sustained intraocular or retinal exposure (Ahmed et al., 2023). Therefore, the development of efficient ocular-targeting modification strategies is critical to achieving precise accumulation of nanozymes in the lesion area (Fig. 4), enhancing bioavailability, and minimizing systemic exposure (Khare et al., 2006; Liu et al., 2024; Stack et al., 2020).

**Fig. 4:**
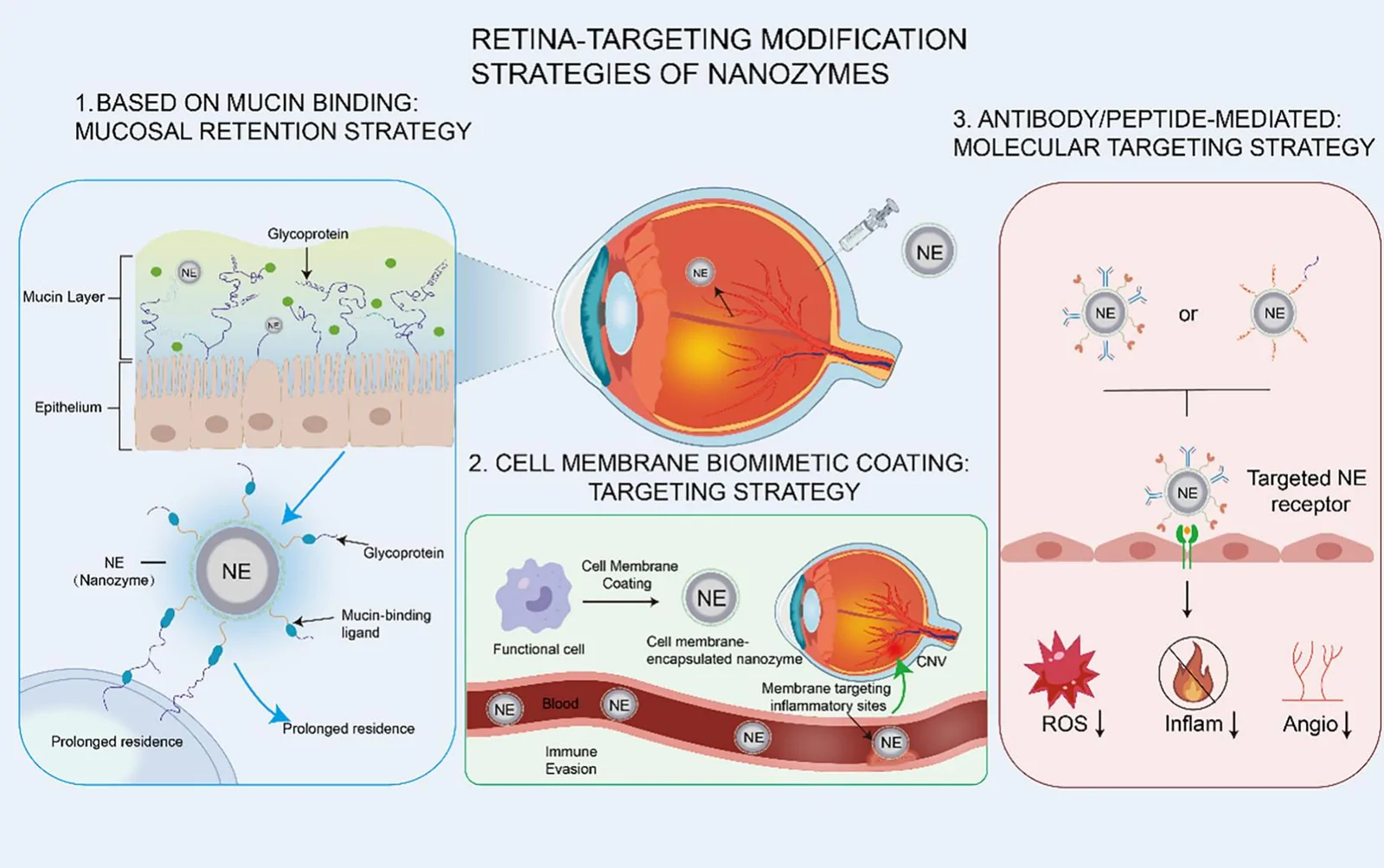
Different targeting modification strategies for nanozymes with different response properties in ocular tissues, including mucin-binding mucosal retention, cell-membrane biomimetic coating and ligand-mediated molecular targeting.

### 3.1 Mucoadhesive strategies based on mucin binding

Mucoadhesion is advantageous for ocular nanozyme delivery, as precorneal residence is typically restricted by tear dilution and drainage. The ocular mucus/glycocalyx contains abundant negatively charged mucins, allowing retention to be enhanced through electrostatic interactions, hydrogen bonding, hydrophobic contacts or more specific covalent/reversible interactions such as thiol-disulfide exchange and boronate ester formation. Cationic polymers such as chitosan can strengthen adhesion, whereas thiolated polymers or phenylboronic acid groups can provide stronger or more selective anchoring. However, greater adhesion does not automatically equate to better delivery, as excessive binding can fix particles in the surface mucus layer and reduce epithelial penetration, while high-viscosity or strong cationic formulations can increase visual blurring, irritation or non-specific membrane interactions. Therefore, a mucoadhesive nanoenzyme system should balance tear resistance, mucus penetration, epithelial uptake and maintaining the accessibility of catalytic sites (Moiseev et al., 2019; Portal et al., 2019).

Zhang et al. developed an iron-doped zeolitic imidazolate framework-derived nanozyme eye drop (PBnZ). By introducing phenylboronic acid groups, reversible boronate esters were formed with cis-diol groups on mucin glycans. These dynamic covalent bonds remained stable under physiological pH conditions, anchoring the nanoparticles within the mucus layer and significantly slowing their clearance by tears. Additionally, the positively charged PBnZ nanoparticles bound to the negatively charged proteoglycan matrix of mucin through electrostatic interactions, further enhancing mucosal adhesion, demonstrating an enhanced therapeutic efficacy (Zhang et al., 2024b).

### 3.2 Ocular microneedle-assisted nanozyme delivery

Microneedle-assisted delivery is mechanistically distinct from mucoadhesion. Rather than relying on surface retention, it bypasses epithelial barriers by creating transient microchannels and deposits the nanozyme directly within or across ocular surface tissues. Liu et al. developed an ocular microneedle system loaded with multienzyme-like nanozymes (MGMN), integrating mucosal retention mechanisms with microneedle delivery technology (Fig. 5). Using hyaluronic acid and poly(methyl methacrylate) as the microneedle matrix, the system incorporated manganese oxide nanocluster-decorated graphdiyne nanosheets (MnO_X_/GDY) as multienzyme-like nanozymes. The carboxyl and hydroxyl groups on the hyaluronic acid chains interacted with non-glycosylated mucin regions through hydrogen bonding and van der Waals forces, while the physical penetration of the microneedles enabled localized and sustained drug release. Compared with eye drops, microneedles can improve local dose efficiency and reduce precorneal loss, but their clinical use requires careful control of insertion depth, mechanical strength, sterility, tissue injury, patient comfort and reproducibility (Liu et al., 2023).

**Fig. 5:**
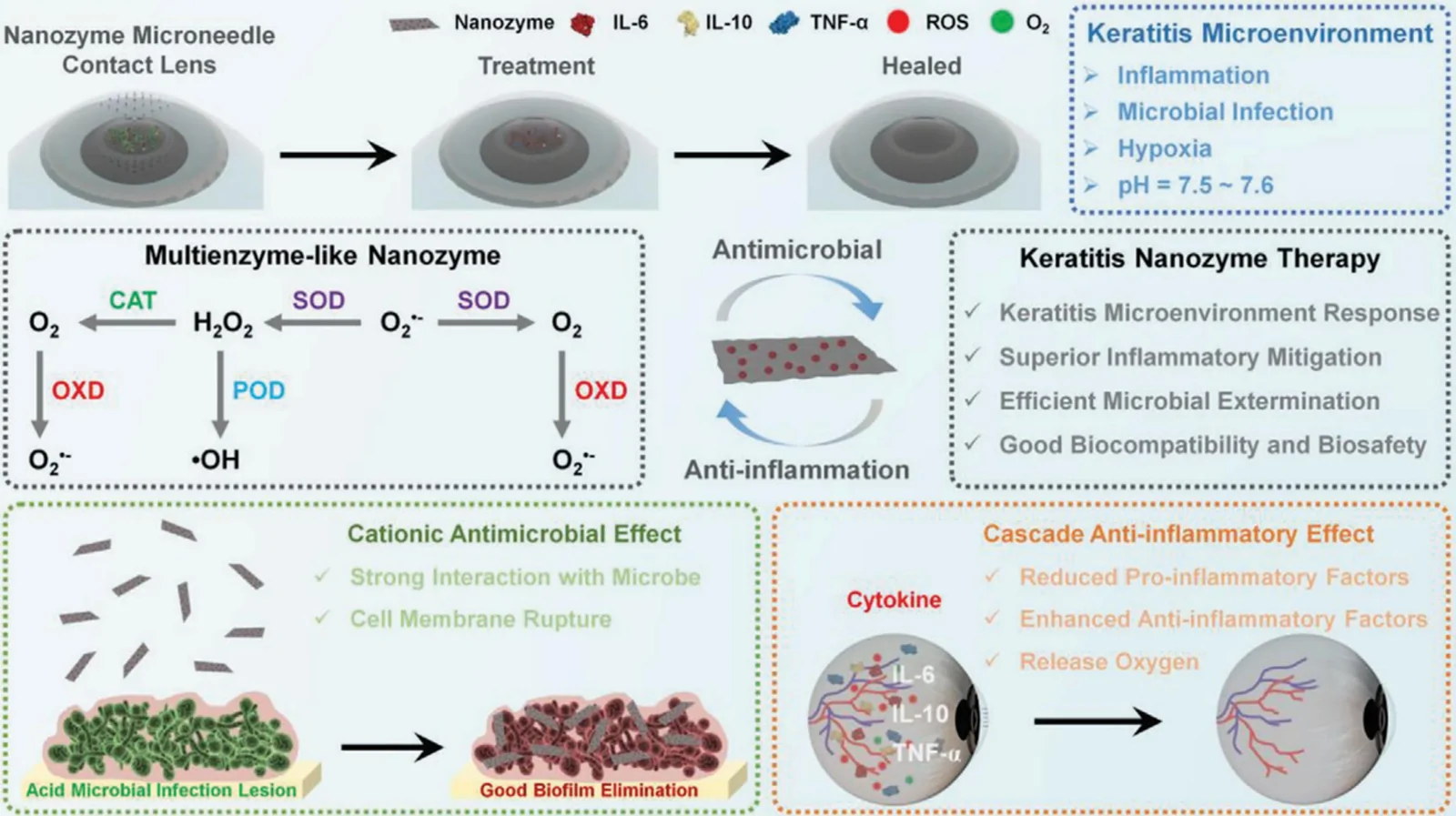
Ocular microneedles loaded with multienzyme-like nanozymes for the treatment of infectious keratitis. This system illustrates a barrier-bypassing strategy in which physical microchannel formation enables localized nanozyme deposition and sustained catalytic action. This figure has been reproduced from ref. Liu et al. (2023) with permission from Wiley, copyright 2023.

### 3.3 Active targeting strategies based on cell membrane biomimetic coating

The core advantage of the cell membrane biomimetic coating strategy lies in constructing a “biological camouflage” nanozyme delivery system by simulating the biological properties of natural cells (Ferreira-Faria et al., 2022; Kroll et al., 2016; Liu et al., 2020; Zhu et al., 2023). Typically, cell membranes derived from cell types that exhibit specific interactions with the retinal lesion microenvironment are selected (Khatoon et al., 2022; Pan et al., 2024; Wang et al., 2026a; Xu et al., 2021). Through physical methods such as extrusion or sonication, the cell membrane is uniformly coated onto the surface of the nanozyme, forming a core-shell structured biomimetic nanocarrier (Liu et al., 2022b; Xie et al., 2024; Zhu et al., 2021). This approach not only preserves the intrinsic enzyme-like catalytic activity of the nanozyme core but also seamlessly transfers functional components (such as specific receptors and adhesion molecules) from the cell membrane to the carrier surface, endowing it with the targeting recognition capabilities and immune evasion properties characteristic of natural cells (Chan et al., 2025; Lei et al., 2022; Marinho et al., 2024).

Hu et al. proposed and validated the concept of “cell membrane-camouflaged nanoparticles”, demonstrating that red blood cell membrane coating significantly enhances the in vivo stability and biocompatibility of nanomaterials, laying a foundation for biomimetic targeted delivery in various disease models (Hu et al., 2011). Feng et al. developed a neutrophil-like cell membrane-coated mesoporous prussian blue nanozyme (MPBzyme@NCM). By coating the surface of the nanozyme with differentiated HL-60 cell membranes, the resulting carrier specifically recognized intercellular adhesion molecule-1 (ICAM-1), which is highly expressed on ischemic cerebral microvascular endothelial cells, and traversed the blood-brain barrier via receptor-mediated transcytosis (Feng et al., 2021).

Despite these advantages, cell membrane coating also introduces manufacturing limitations that can become more pronounced during clinical scale-up. Membrane preparation generally requires cell expansion, harvesting, lysis, repeated centrifugation or purification, vesicle formation and subsequent nanoenzyme coating. Material loss during membrane isolation and nanoparticle extrusion or filtration can reduce membrane yield, while donor source, cell passage, activation state and culture conditions may alter membrane protein and lipid composition, leading to batch-to-batch variations. Traditional extrusion is effective on a laboratory scale, but it may be time-consuming and difficult to scale up (Zhang et al., 2021; Zhang et al., 2024a).

Membrane coating completeness also require critical evaluation. Although extrusion may favor a right-side-out membrane configuration, current preparation methods cannot ensure uniform leaflet orientation or complete coverage across all particles. Incomplete coating or improperly oriented membranes can expose normal intracellular domains or mask extracellular targeted proteins, potentially changing receptor recognition, macrophage interactions, and immune evasion behavior. In addition, the standardization of cell provenance, preservation of functional membrane proteins during processing and storage, and acceptable batch-to-batch variability also create additional regulatory burdens. Therefore, future biomimetic ocular nanozymes should incorporate quantitative analysis of membrane yield, coating integrity, orientation and functional-protein retention, together with scalable and standardized coating processes, rather than merely relying on particle size and electron microscopy to establish successful membrane camouflage. Importantly, the integrity of biomimetic membrane coatings after traversing ocular barriers remains insufficiently characterized. Future studies should directly evaluate whether membrane coverage, orientation, and key functional proteins are retained after corneal or retinal barrier transport and during subsequent intraocular residence, as structural disruption of the coating may compromise targeting and immune-evasion functions.

### 3.4 Antibody/Peptide-mediated molecular targeting strategies

The core mechanism of antibody/peptide-mediated molecular targeting strategies is based on specific ligand-receptor interactions (Lin et al., 2022; Sasahara et al., 2023; Shao et al., 2024). The process primarily involves three key steps: first, targeted nanozymes in circulation bind to specific receptors overexpressed on the surface of vascular endothelial cells or lesional cells at the site of retinopathy via ligands, achieving initial targeted enrichment; second, they traverse the compromised blood-retinal barrier through receptor-mediated endocytosis or transcytosis to enter the retinal tissue; third, they maintain their enzyme-like activity within the pathological microenvironment, precisely scavenging ROS, suppressing inflammation, or modulating vascular proliferation to exert therapeutic effects (Novotny et al., 2020; Park et al., 2021). Compared with passive targeting, this active targeting strategy significantly enhances therapeutic specificity and efficacy while reducing off-target toxicity. Zhang et al. developed a CD38-targeting peptide-modified nanocarrier that specifically bound to CD38 receptors, enabling precise targeted delivery of bortezomib. This approach not only significantly improved drug accumulation efficiency in the lesion area but also enhanced biocompatibility through red blood cell membrane camouflage (Zhang et al., 2023a).

### 3.5 Stimulus-responsive drug release strategies

The microenvironment of retinopathy exhibits significant specific characteristics, including abnormally elevated ROS, a localized decrease in pH, and enhanced activity of various pathologically relevant enzymes. These biochemical signals provide natural targets for the construction of precision drug delivery systems (Sorrentino et al., 2016; Wang et al., 2026b; Yan et al., 2023). Stimulus-responsive drug release strategies utilize nanocarriers designed to be sensitive to specific stimuli within the retinopathic microenvironment, enabling targeted activation and controlled release of nanozymes at the lesion site (Ko et al., 2026; Zhang and Tong, 2023). This approach ensures the stability of nanozymes during blood circulation while precisely amplifying therapeutic effects in the pathological area, thereby significantly reducing damage to normal retinal tissue (Liu et al., 2025; Wen et al., 2020). This section will focus on three aspects: ROS-responsive drug release strategies, pH-responsive drug release strategies, and enzyme-responsive drug release strategies.

#### 3.5.1 ROS-responsive drug release strategies

As a metabolically highly active neural sensory tissue, the retina is characterized by excessive accumulation of ROS resulting from mitochondrial dysfunction, which constitutes a core pathological mechanism in diseases such as diabetic retinopathy and age-related macular degeneration (Klein et al., 2018; Maruko et al., 2016; Wu et al., 2024a). ROS not only directly damage the membrane structures of retinal vascular endothelial cells and photoreceptors, leading to cell death and functional loss, but also activate signaling pathways such as PI3K/AKT and NF-κB, triggering inflammatory cascades and pathological neovascularization, thereby exacerbating retinal damage and functional degeneration (Böhm et al., 2023; Xie et al., 2025). Consequently, targeted ROS scavenging and modulation of oxidative stress have become critical strategies for the treatment of these diseases (Mi et al., 2025).

ROS-responsive drug delivery systems typically incorporate ROS-sensitive chemical bonds into nanocarriers, enabling bond cleavage or structural disassembly under high oxidative stress conditions, thereby greatly enhancing therapeutic precision and efficiency (Wu et al., 2024b; Zhang et al., 2025c). Zhou et al. developed a HOLN-NPs nanprodrug system, in which nicotinamide and oleic acid were conjugated via a thioketal bond to form OLN monomers (Fig. 6). These monomers subsequently underwent self-assembly and multimicellar aggregation to form HOLN-NPs. In the high-ROS environment of glaucomatous lesions, the thioketal bond undergoes cleavage, triggering the synergistic release of nicotinamide and oleic acid. Nicotinamide improves mitochondrial function by inhibiting the Cyt C/PARP signaling pathway, while oleic acid activates the CaMKII/CREB signaling axis. Together, they exert protective effects against retinal ganglion cell apoptosis and axonal damage, demonstrating the advantages of ROS-responsive nanodelivery systems in multi-target synergistic therapy (Zhou et al., 2024).

**Fig. 6:**
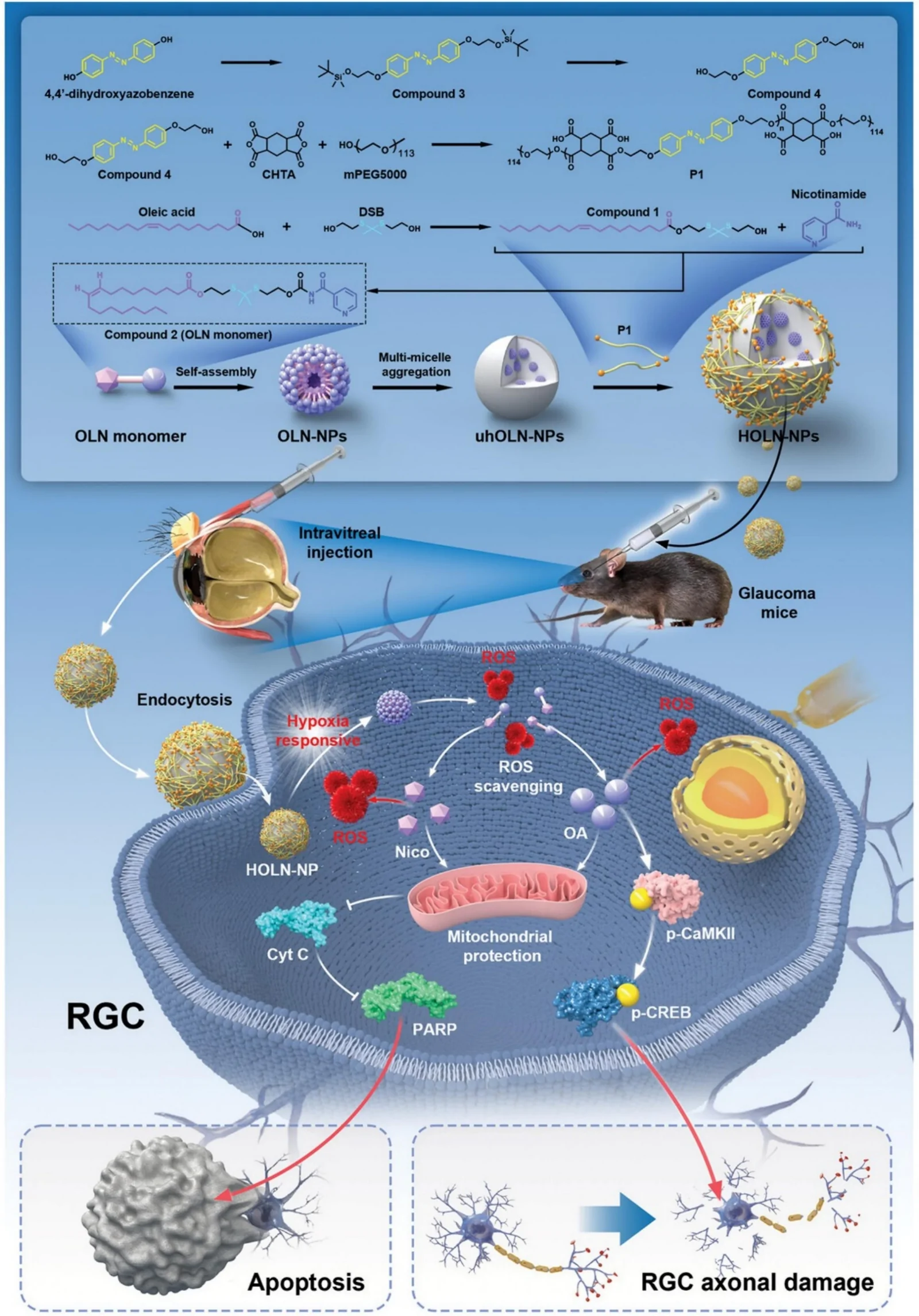
Schematic illustration of ROS-responsive HOLN-NPs for protection of retinal ganglion cells. This design couples pathological ROS-triggered release with mitochondrial protection and neuroprotection, achieving multiple therapeutic effects. This figure has been reproduced from ref. Zhou et al. (2024) with permission from Wiley, copyright 2024.

#### 3.5.2 pH-responsive drug release strategies

During the progression of retinopathy, retinal ischemia-hypoxia, inflammatory infiltration, and metabolic reprogramming are often accompanied by lactate accumulation and localized acidification. This “lesional microacidity” not only affects the expression of angiogenesis-related factors but also provides an exploitable endogenous trigger signal for targeted drug delivery (Li et al., 2016; Zhou and Du, 2026). The core principle of pH-responsive drug release strategies lies in utilizing pH-sensitive functional materials to construct nanocarriers that remain stable under normal physiological pH (ocular surface pH of approximately 7.4) but undergo rapid structural or physicochemical changes (such as dissolution, swelling, charge reversal, or bond cleavage) upon reaching the acidic lesion microenvironment, thereby enabling precise and rapid drug release (Teng et al., 2026). Nguyen et al. constructed a pH-responsive cerium oxide nanocapsule delivery system utilizing the reversible protonation of amino groups on chitosan chains. Under acidic conditions (pH 5.5), the amino groups of chitosan become protonated, leading to enhanced electrostatic repulsion between polymer chains, which causes swelling of the coating layer and promotes the release of pilocarpine from the nanocapsules. When the pH of the system is adjusted to neutral (pH 7.4), chitosan undergoes deprotonation, electrostatic repulsion diminishes, and the coating contracts, forming a dense sealing layer on the nanocarrier surface that effectively encapsulates the drug within the nanocapsules and prevents premature leakage (Nguyen et al., 2022).

#### 3.5.3 Enzyme-responsive drug release strategies

In the pathological microenvironment of fundus retinopathy, the aberrant overexpression of various hydrolases and matrix-degrading enzymes represents an important pathological hallmark (Rapalli et al., 2019). For instance, during diabetic retinopathy, due to ischemia-reperfusion injury and neovascularization, the activities of matrix metalloproteinases, hyaluronidase, phospholipase, and other hydrolases are significantly elevated, providing exploitable endogenous signals for the design of enzyme-responsive nanocarrier-based targeted drug delivery (Eyush et al., 2024; Fouda et al., 2022). Enzyme-responsive systems rely on lesion-specific enzyme-mediated bond cleavage or material degradation to achieve targeted drug release, controlled release, and reduced toxicity to non-target tissues. Huang et al. chemically conjugated graphene oxide quantum dots to an octadecyl-modified MMP9 substrate peptide sequence, which can be specifically cleaved by activated MMP9. In the lesion area, overexpressed MMP9 recognizes and cleaves this sensitive peptide bond, triggering the controlled release of the encapsulated minocycline and the graphene oxide quantum dots, which possess antioxidant and anti-inflammatory activities (Huang et al., 2023).

#### 3.5.4 Comparison of different stimulus-responsive drug release strategies

Although ROS-responsive, pH-responsive, and enzyme-responsive drug release strategies each demonstrate significant advantages in the precision treatment of fundus retinopathies, substantial differences exist among these stimulus-responsive mechanisms in terms of applicable scenarios, response precision, and clinical application potential (Table 2).

**Table 2:** Design strategies, advantages and disadvantages, and clinical applications in the eye of different responsive nanozyme drug delivery systems.

Response type	Responding mechanism	Design strategy	Advantage	Boundedness	Applicable scene
ROS-responsive	ROS-sensitive bonds (thioketal, thioether or peroxide-containing linkages) undergo cleavage or structural change under elevated oxidative stress, triggering drug release.	Thioketal/thioether linkages, peroxide-sensitive groups and ROS-responsive carrier architectures.	It responds quickly and dynamically, can achieve multi-targeted coordinated treatment, and is applicable to diseases related to oxidative stress.	The ROS levels vary greatly among individuals, which may cause the non-targeted regions to be released prematurely, thereby affecting the accuracy.	Diseases such as diabetic retinopathy and ischemia-reperfusion injury.
pH-responsive	Utilizing pH-sensitive materials, which undergo structural changes (such as expansion and charge reversal) in the acidic lesion microenvironment, to trigger the release of drugs.	Using pH-sensitive materials (such as chitosan), the reversible protonation property of these materials is utilized to achieve controlled release of drugs.	The response conditions are clear and the design is mature, and the controllability is good in the local ocular drug delivery system.	The pH variation range is relatively mild, and a high threshold for material acid sensitivity is required; a single pH trigger may not be sufficient to respond in a complex environment.	The lesion area characterized by retinal ischemia and hypoxia, inflammatory infiltration, and accumulation of lactic acid.
Enzyme-responsive	The precise release is achieved by relying on disease-specific enzymes (such as MMP9) to mediate the breaking of chemical bonds or the degradation of materials.	By designing enzyme-sensitive substrate peptide sequences, they can be broken down under the action of specific enzymes, thereby achieving drug release.	High selectivity, enabling precise molecular-level release, is suitable for pathological stages where enzyme activity significantly increases.	Based on the accurate analysis of the enzyme expression profile, there is a significant individual variation in enzyme activity, which may affect the stability of the treatment.	The stage of active diabetic retinopathy, neovascularization and tissue remodeling.

ROS-responsive systems are highly dependent on markedly elevated oxidative stress levels in the lesion area, enabling rapid and dynamic responses to the pathological region. They are particularly suitable for diseases characterized by oxidative imbalance, such as diabetic retinopathy and ischemia-reperfusion injury (Hao et al., 2020; Yu et al., 2022). However, a potential limitation is that ROS levels exhibit considerable heterogeneity across different disease stages and among individuals. Overly sensitive ROS-responsive materials may undergo premature release in non-target areas with mild inflammatory activation, thereby compromising therapeutic precision.

In contrast, pH-responsive drug delivery systems rely on the relatively stable acidification characteristics of the lesion microenvironment, offering advantages such as well-defined response conditions and mature design strategies, demonstrating good controllability in ophthalmic topical administration systems. Nevertheless, the magnitude of pH changes in retinopathy lesions is typically modest, imposing higher demands on the precise tuning of acid-sensitivity thresholds for these materials (Guo et al., 2023). Furthermore, a single pH-trigger mechanism may face insufficient responsiveness in complex pathological environments.

Enzyme-responsive drug release strategies leverage the high selectivity of lesion-specific enzymes, providing a unique pathway to achieve “molecular-level precision release”. They are particularly suitable for pathological stages characterized by active neovascularization and tissue remodeling. However, the design of such systems is highly dependent on accurate profiling of disease-associated enzyme expression patterns. Dynamic variations in enzyme activity among different patients and across disease stages may pose challenges to the therapeutic stability and universal applicability of these systems.

Notably, current research trends are progressively shifting from single stimulus-responsive systems toward multi-stimulus synergistic response strategies, such as integrating ROS/pH or ROS/enzyme dual-trigger mechanisms, to enhance the adaptability of nanozyme delivery systems within the complex retinopathy microenvironment (Tong et al., 2023). In the future, fine-tuning of stimulus-response thresholds combined with the physiological characteristics of ocular administration routes is expected to further promote the clinical translation of stimulus-responsive nanozyme systems.

### 3.6 Ocular penetration enhancement strategies

Penetration enhancers provide a conceptually distinct strategy from mucoadhesion. Instead of primarily prolonging precorneal residence, they transiently increase permeability across ocular epithelia. Classical ocular penetration enhancers include chelating agents such as EDTA, surfactants, bile salts, cyclodextrins and cell-penetrating peptides. Their mechanisms include reversible loosening of epithelial tight junctions, membrane fluidization, altered partitioning and improved solubilization. However, deliberate disruption of the tear film or epithelial barrier can cause irritation and chronic toxicity, particularly during repeated treatment. Therefore, an ideal ophthalmic enhancer should act rapidly and reversibly at a low concentration without causing damage (Santos et al., 2025).

Wu et al. demonstrated that TAT-functionalized liposomes increased transcorneal penetration and aqueous humor exposure by promoting electrostatic interaction with corneal epithelial cells and partially modulating tight junctions (Wu et al., 2021). More recently, Sun et al. combined penetratin with hyaluronic acid-modified liposomes for topical delivery of conbercept to the posterior segment. This formulation reached the retina via corneal and non-corneal pathways and produced an intraocular conbercept concentration 11.55-fold higher than unmodified drug after topical administration (Sun et al., 2024a). These studies illustrate two transferable design principles for ophthalmic nanozymes, including cell-penetrating surface ligands and dual penetration/targeting coatings.

Accordingly, corneal and conjunctival barrier recovery, epithelial integrity, stimulation, tear film effect, local concentration-time distribution, and target tissue exposure should be evaluated during using penetration-enhanced ophthalmic nanozymes. Combining controlled mucoadhesion with a mild and reversible permeable enhanced surface may be more preferable than maximizing retention or epithelial destruction alone.

### 3.7 Ophthalmic pharmacokinetics and clearance of nanozymes

In addition to catalytic activity and molecular targeting, the therapeutic performance and biosafety of ophthalmic nanozymes largely depend on their ophthalmic pharmacokinetic behavior. Particle size, surface charge, colloidal stability and surface functionalization collectively determine diffusion through ocular barriers, cellular uptake, clearance routes and residence time (Bairagi et al., 2025). Therefore, these physicochemical parameters should be considered together with catalytic selectivity when designing nanozyme-based ophthalmic drug delivery systems.

Following topical administration, nanozymes are exposed to rapid tear turnover, blinking, mucus-mediated entrapment and nasolacrimal drainage. Cationic surface can electrostatically interact with negatively charged ocular mucin, increasing precorneal retention and, in some cases, cellular uptake. However, excessive positive charge may produce strong mucus binding, restrict penetration through the mucus layer, and increase non-specific membrane interactions. In contrast, hydrophilic or near-neutral coatings can reduce non-specific adhesion and promote mucus penetration, although weakly adherent particles may also be removed through tear drainage (Popov, 2020). Thus, topical nanozyme design requires a balance among mucoadhesion, mucus penetration and precorneal clearance rather than maximization of surface charge alone.

The particle size of nanozyme and whether they carry a positive or negative charge can produce different effects when administered through the vitreous. The vitreous is a hydrated extracellular matrix containing collagen fibrils, hyaluronic acid and soluble macromolecules, thus serving as a barrier for space and electrostatic diffusion. Smaller, non-aggregated particles generally diffuse more readily than larger or aggregated structures, whereas strongly cationic nanoparticles can interact with negatively charged vitreous components and become partially immobilized. Single-particle tracking experiments have revealed that cationic nanoparticles exhibit markedly restricted mobility in intact vitreous, whereas PEGylation substantially enhances their mobility by reducing adhesive interactions and aggregation (Martens et al., 2013). Thus, increasing retention in the vitreous through strong charge binding may not be helpful, as long-term local retention might make the nanozymes distribute poorly in the retina.

Nanozymes in the vitreous can be eliminated through anterior and posterior pathways. Anterior clearance involves diffusion toward the anterior chamber followed by aqueous turnover and trabecular or uveoscleral outflow, whereas posterior elimination requires transport across retinal tissues and the blood-retinal barrier toward the choroidal circulation (del Amo et al., 2016; Huang and Chau, 2019). Particle size, surface charge and interaction with extracellular matrices (ECMs) influence the relative contributions of these pathways. Highly mobile ultra-small nanostructures can be rapidly distributed, but they can also be more easily removed, while larger or strongly interacting particles can display longer apparent residence times at the expense of homogeneous retinal exposure. Therefore, residence time should be interpreted together with retinal distribution and local concentration-time profiles, rather than as an isolated indicator of delivery efficiency. Cellular sequestration represents an additional determinant of intraocular disposition. Hyalocytes are resident macrophage-like phagocytes on the vitreous that can engulf foreign particles, while retinal microglia and infiltrating macrophages may also participate in nanoparticle recognition and uptake, especially under inflammatory conditions (Boneva et al., 2024). Hydrophilic, PEGylated, zwitterionic or biomimetic surface coatings can reduce non-specific protein adsorption and phagocyte recognition, thereby prolonging intraocular duration. However, excessive immune evasion can simultaneously reduce uptake by therapeutic target cells. Ligand-mediated targeting can partly resolve this issue by limiting non-specific sequestration while retaining receptor-specific cellular internalization.

Ocular nanozyme design involves an inherent pharmacokinetic trade-off. For topical administration, sufficient mucosal retention must be coordinated with epithelial penetration. For intravitreal administration, vitreous diffusion and retinal pathways must be balanced against premature clearance and long-term accumulation. Future studies should report hydrodynamic diameter, zeta potential, colloidal stability in ocular fluids, vitreous diffusion behavior, ocular concentration-time profiles, cell type-specific localization and route-specific clearance whenever possible. Such quantitative pharmacokinetic characterization is particularly important for persistent inorganic nanozyme, as prolonged catalytic activity is highly attractive in treatment, but it may also increase the risk of long-term tissue accumulation and off-target effects.

## 4 Applications of nanozymes in ROS-related ophthalmic diseases

Nanozymes exhibit unique advantages in the treatment of ROS-related ophthalmic diseases by virtue of their enzyme-like activities and engineerable structures. On one hand, nanozymes can continuously scavenge ROS and modulate redox homeostasis within pathological microenvironments characterized by high oxidative stress, chronic inflammation, and metabolic imbalance (Zhu et al., 2025b). On the other hand, their surfaces can be functionalized to achieve targeted delivery to retinal pigment epithelium, retinal vascular endothelial cells, inflammatory cells or corneal epithelium, thereby enhancing local effective concentrations while reducing the risk of systemic exposure. The following sections summarize nanozyme applications in age-related macular degeneration, diabetic retinopathy, dry eye disease and glaucoma.

### 4.1 Age-related macular degeneration

Age-related macular degeneration is the leading cause of irreversible central vision impairment in the elderly population worldwide (Fleckenstein et al., 2024; Guymer and Campbell, 2023). Its pathological changes primarily involve the retinal pigment epithelium, Bruch's membrane, and the choriocapillaris layer. The onset and progression of AMD result from the interplay of genetic susceptibility, environmental factors and age-related molecular damage, among which oxidative stress is widely recognized as one of the core pathological drivers of disease progression. Current standard therapy involves intravitreal injection of anti-VEGF agents. However, challenges still remain, including the need for repeated intraocular injections and suboptimal responses in some patients (Falcão et al., 2025; Kushwah et al., 2023; Ruan et al., 2021). Nanozymes, leveraging their antioxidant, anti-inflammatory, excellent stability and multi-enzyme synergistic properties, demonstrate unique advantages in modulating the complex pathological processes of AMD.

Chronic blue light exposure and lipofuscin accumulation in retinal pigment epithelium cells lead to excessive ROS generation, which not only directly damages mitochondrial membrane potential and triggers apoptosis but also upregulates the secretion of inflammatory cytokines such as TNF-α and IL-6 through activation of the NLRP3 inflammasome, ultimately resulting in drusen deposition and retinal structural disruption. In wet AMD, choroidal neovascularization is the primary cause of visual impairment (Chen et al., 2023; Ozawa, 2020). Cai et al. designed the MVPC nanoformula (Fig. 7). By loading V_2_C quantum dots with multiple enzymatic activities and simultaneously simulating six enzymes including superoxide dismutase and catalase, it effectively neutralizes excessive ROS and blocks oxidative stress-induced endothelial activation pathways. After co-loading this nanozyme with IL-10 plasmids into a biomimetic metal-organic framework and modifying it with mesenchymal stem cell membrane vesicles, the system achieved targeted controlled release through pH-responsive degradation while enhancing corneal retention and cellular uptake efficiency via the membrane vesicles. In a rat model of Corneal neovascularization, this formulation significantly inhibited neovascularization and reduced inflammatory cell infiltration without inducing obvious biotoxicity (Cai et al., 2025). Sun et al. developed polyethylene glycol-coated rhodium nanozymes (PEG-RhZ), which not only possess potent scavenging capabilities against ROS and reactive nitrogen species but also effectively protect retinal pigment epithelium barrier function by inhibiting microglial activation and downstream pro-inflammatory factors, ultimately reducing photoreceptor apoptosis and improving visual function in the later stages. Collectively, these findings suggest that nanozyme offers a promising disease-modifying strategy for AMD through combined antioxidant and anti-inflammatory effects (Sun et al., 2024b).

**Fig. 7:**
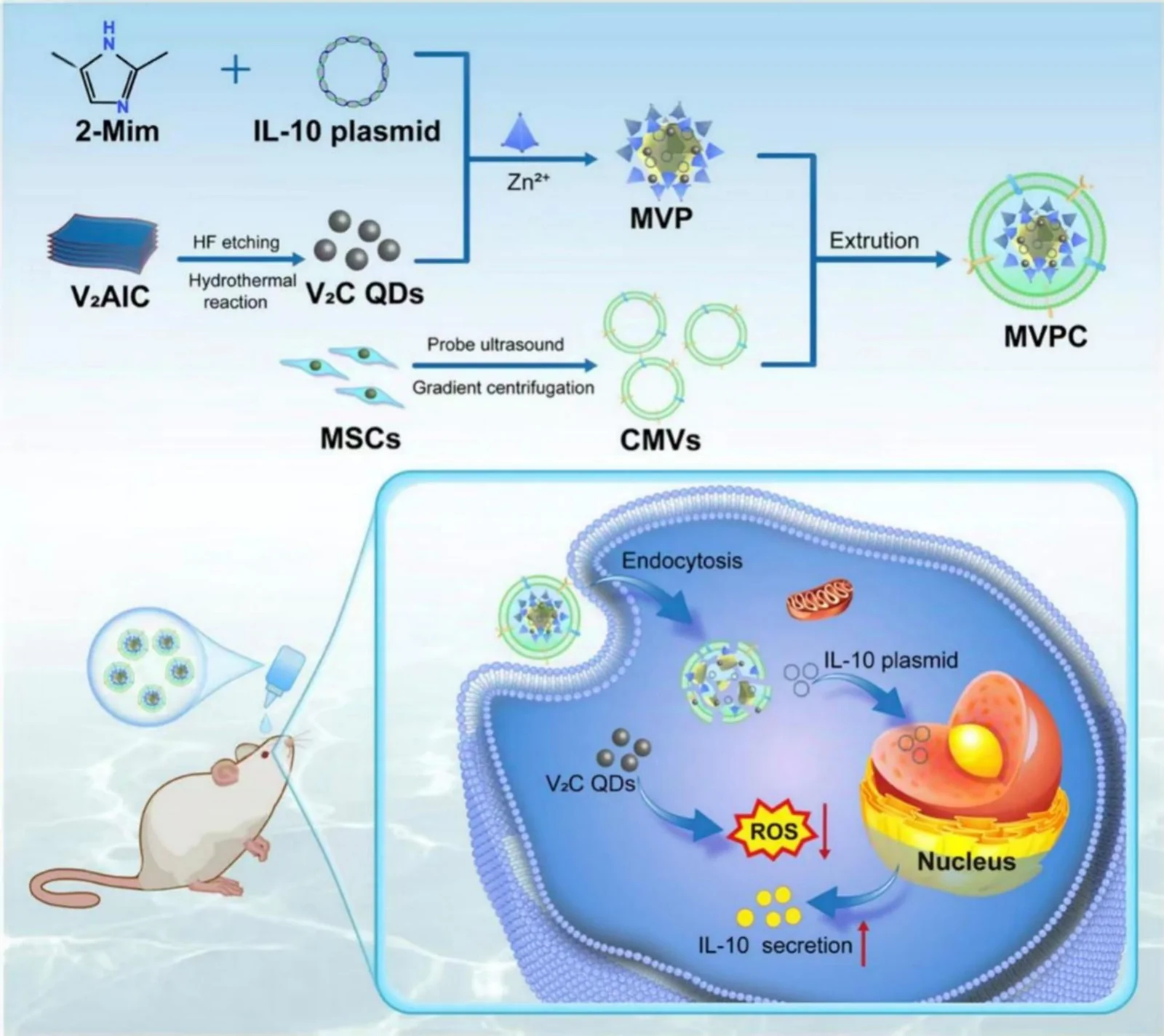
Schematic illustration of MVPC nanotherapeutics for synergistic CNV therapy. This platform integrates catalytic ROS regulation, IL-10-mediated immunomodulation, biomimetic membrane functions and pH-responsive release, illustrating the benefits and complexity of multicomponent nanozyme systems. This figure has been reproduced from ref. Cai et al. (2025) with permission from Wiley, copyright 2025.

### 4.2 Diabetic retinopathy

Diabetic retinopathy (DR) is one of the most common and severe microvascular complications of diabetes mellitus and has become the leading cause of blindness among the working-age population worldwide (Roy et al., 2016). Its pathological progression is complex, originating from chronic hyperglycemia-induced metabolic disturbances that ultimately lead to progressive structural and functional damage to the retinal microvasculature. The core pathological mechanism involves a multifaceted vicious cycle: chronic hyperglycemia triggers the activation of the polyol pathway, protein kinase C pathway and accumulation of advanced glycation end products, ultimately resulting in mitochondrial dysfunction and excessive production of ROS (Calderon et al., 2017). This sustained oxidative stress state represents the initiating and central event in the pathogenesis of DR, directly damaging retinal capillary pericytes and endothelial cells, disrupting the blood-retinal barrier, and causing vascular leakage and retinal edema. Concurrently, oxidative stress, together with hypoxia, drives the overexpression of pro-angiogenic factors such as pathological vascular endothelial growth factor, promoting retinal neovascularization and marking the progression of the disease into the vision-threatening proliferative stage (Malaviya et al., 2024). Furthermore, the chronic inflammatory response triggered by oxidative stress, along with the subsequent activation of glial cells, further exacerbates damage to the neurovascular unit and retinal neurodegeneration (Fazendeiro et al., 2025).

Currently, clinical treatment of DR primarily relies on anti-VEGF agents, laser photocoagulation and corticosteroids. While these therapeutic approaches have achieved certain efficacy in inhibiting neovascularization and alleviating macular edema, their targets are mainly focused on downstream pathological events of the disease, making it difficult to effectively intervene in the early pathological processes dominated by oxidative stress and chronic inflammation. Moreover, the need for frequent intravitreal injections poses challenges to patient compliance, and the potential side effects associated with long-term administration, such as elevated intraocular pressure and infection risk, further limit the widespread application of conventional treatment strategies (Everett and Paulus, 2021). Therefore, the development of novel therapeutic approaches capable of modulating oxidative stress and inflammatory responses at the pathological origin has become a critical direction requiring breakthroughs in the field of DR treatment.

Among these, cerium oxide nanozymes, owing to their reversible Ce^3+^/Ce^4+^ conversion property, can simultaneously scavenge superoxide anions and hydrogen peroxide, and have been widely utilized to alleviate DR-associated oxidative stress and inflammatory responses (Liu et al., 2021c; Meng et al., 2025). Relevant studies have demonstrated that this class of nanozymes effectively protects retinal vascular structure, improves neuronal function, and to some extent inhibits pathological neovascularization. Crystal S. Shin et al. delivered cerium oxide nanoparticles to the retina using a non-invasive ceramic wafer. Following treatment, they observed significantly reduced signals of the ROS marker 8-hydroxy-2′-deoxyguanosine and the fluorescent probe dichlorofluorescein in the retina of vldlr (−/−) mice, along with markedly downregulated expression of VEGF protein in the retina. These findings indicate that cerium oxide nanoparticles effectively scavenge excessive ROS, block VEGF upregulation, and suppress subsequent inflammatory responses and neovascularization (Shin et al., 2022).

### 4.3 Dry eye disease

Dry eye disease (DED) is a chronic ocular surface disorder caused by tear film homeostasis imbalance. Its core pathological mechanisms can be summarized as tear film instability, hyperosmolarity-induced damage, ocular surface inflammation, and neurosensory abnormalities (Clayton, 2018). Oxidative stress is recognized as one of the upstream driving factors in DED. Excessive ROS directly induce apoptosis and disruption of tight junctions in corneal and conjunctival epithelial cells and promote phosphorylation of the transcription factor NF-κB, leading to the release of inflammatory cytokines such as interleukin-1β and interleukin-18. Concurrently, ROS activate the NLRP3 inflammasome, further promoting the secretion of IL-1β and IL-18, thereby exacerbating ocular inflammation. This cascade leads to goblet cell depletion, insufficient mucin secretion and decreased tear film stability. Moreover, ROS directly damage cellular components including lipids, proteins and DNA, triggering inflammatory responses required for cellular repair. Therefore, as an upstream signal for inflammation, ROS represents a critical target for regulation of DED inflammation (Hu et al., 2025).

Conventional therapies, such as artificial tears, cyclosporine A and corticosteroids, only provide symptomatic relief for ocular surface discomfort and are accompanied by side effects. Due to the inability of ocular surface drugs to achieve long-term retention, they require frequent administration at high concentrations, offering only short-term efficacy without sustained symptom relief, and lack significant intervention for ROS scavenging, which is a crucial step in the pathological cascade. Utilizing nanozymes with multi-enzyme-like activities to continuously scavenge ROS, suppress inflammation and promote epithelial repair is considered a potential disease-modifying strategy distinct from conventional therapies.

Chu et al. developed a diatomic nanozyme based on iron and manganese bimetallic single atoms embedded in a nitrogen-doped carbon matrix and modified with hydrophilic polymers. Its core mechanism involves precise upstream inhibition of the ROS/NLRP3 signaling axis, effectively blocking NLRP3 inflammasome activation, thereby significantly downregulating the expression of downstream pro-inflammatory cytokines IL-1β and IL-18 while suppressing corneal epithelial cell apoptosis. Animal experiments confirmed that this diatomic nanozyme eye drop effectively alleviated ocular surface inflammation, promoted corneal epithelial repair, restored goblet cell density and increased tear secretion, thereby intervening at multiple points and breaking the vicious cycle of DED (Chu et al., 2024).

Bao et al. developed a virus-inspired nanozyme that achieves efficient ocular surface penetration and long-term ferroptosis inhibition through a biomimetic strategy (Fig. 8). This nanozyme mimics the surface topology of the rabies virus and the function of the RVG29 protein, enabling specific binding to nicotinic acetylcholine receptors on corneal epithelial cells and promoting receptor-mediated endocytosis, thereby significantly enhancing cellular uptake efficiency. The cyclosporine A loaded within its core acts synergistically with mesoporous cerium oxide nanozymes exhibiting Ce^3+^/Ce^4+^ valence cycling to scavenge up to 95% of ROS and restore intracellular glutathione levels, maintaining a ferroptosis inhibition rate of 90% even under continuous oxidative stress stimulation (Bao et al., 2025). Additionally, this system modulates macrophage polarization from the M1 to M2 phenotype, improving the ocular surface immune microenvironment. This dual strategy integrating penetration enhancement with ferroptosis inhibition offers a novel approach with significant clinical translation potential for the treatment of DED.

**Fig. 8:**
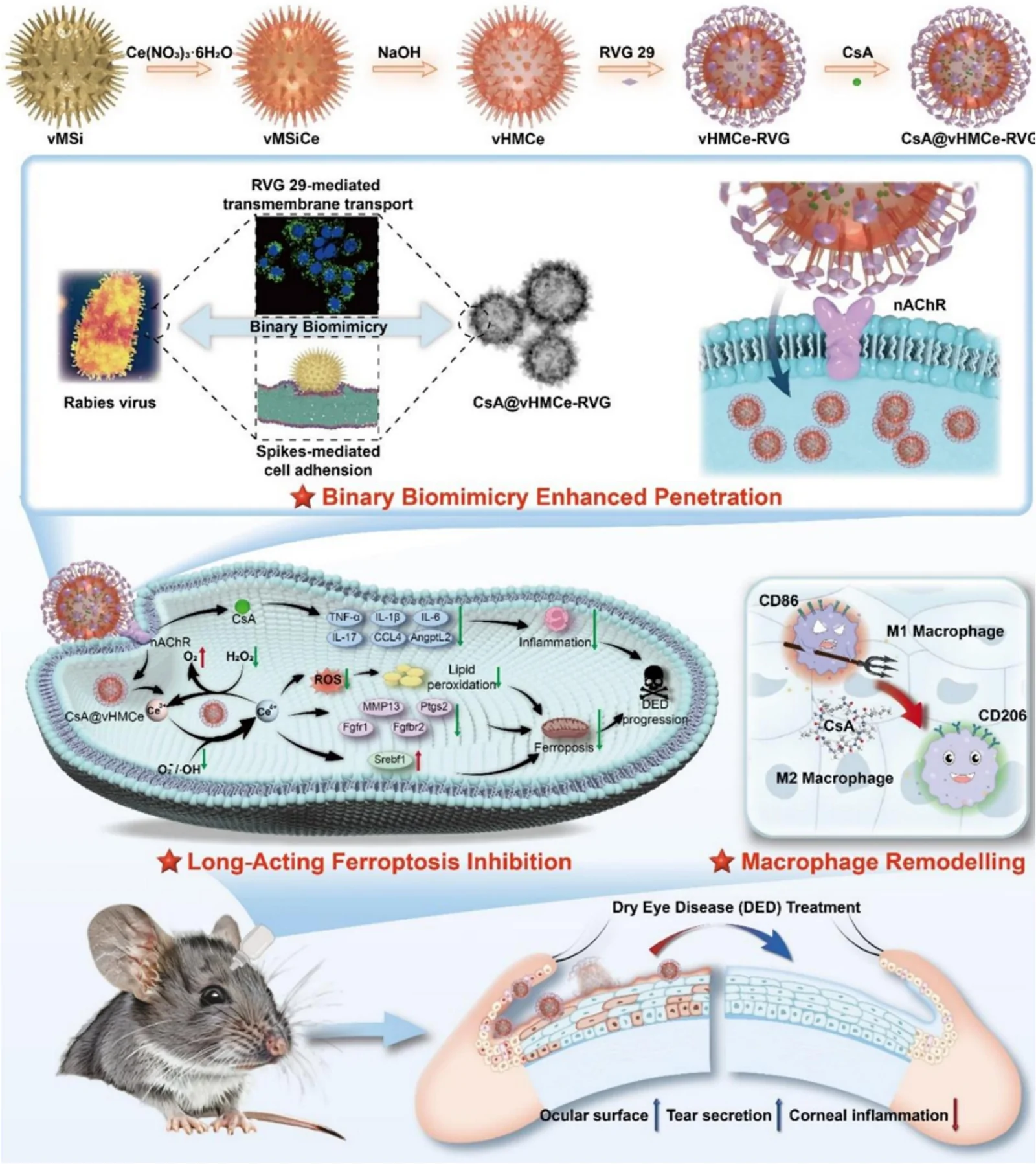
Schematic illustration of virus-mimicking cerium oxide nanozymes for DED. This design combines receptor-assisted epithelial penetration, Ce^3+^/Ce^4+^-dependent antioxidant catalysis, ferroptosis inhibition and macrophage remodeling, highlighting the coupling of delivery and microenvironment regulation. This figure has been reproduced from ref. Bao et al. (2025) with permission from Elsevier, copyright 2025.

### 4.4 Glaucoma

Glaucoma is a leading cause of irreversible blindness worldwide and is characterized by progressive retinal ganglion cell apoptosis and irreversible visual impairment. Its major risk factors include elevated intraocular pressure, oxidative stress and neuroinflammation (Jonas et al., 2017; Salem et al., 2018). Under conditions of high intraocular pressure or insufficient blood supply to the optic nerve, retinal tissues (particularly RGCs and their axons) exhibit dramatically increased energy demands, leading to dysfunction of the mitochondrial electron transport chain and excessive ROS production. Excess ROS not only directly damages mitochondrial DNA and membrane lipids in RGCs, causing energy metabolism disruption and axonal transport impairment, but also activates microglia and promotes their polarization toward the pro-inflammatory M1 phenotype, releasing inflammatory mediators such as TNF-α and IL-6, which further exacerbate neuronal apoptosis (Zhang et al., 2023b). Therefore, effectively eliminating excessive ROS is also a key strategy for glaucoma treatment.

Conventional therapies primarily manage intraocular pressure through antihypertensive medications or surgical interventions such as filtration surgery. However, even when intraocular pressure is controlled, some patients continue to experience progressive optic nerve damage and visual field defects, and these therapies demonstrate limited efficacy in normal-tension glaucoma (Casson, 2022). Given that conventional approaches lack effective intervention against localized high oxidative stress and microenvironmental inflammation in target tissues, they cannot fundamentally halt ROS-driven optic nerve degeneration. Consequently, there is an urgent clinical need to develop disease-modifying strategies that directly scavenge ROS and provide neuroprotection.

Luo et al. prepared hollow cerium oxide nanoparticles with varying shell thicknesses. Their findings demonstrated that cerium oxide nanoparticles with a shell thickness of 20 nm achieved sustained release of the ROCK inhibitor Y-27632, extending the duration of drug action to 10 days and significantly prolonging therapeutic efficacy compared with conventional formulations. Beyond serving as a drug reservoir, cerium oxide itself possesses intrinsic reactive oxygen species scavenging capacity, mimicking catalase and superoxide dismutase activities to synergistically counteract oxidative stress associated with glaucomatous neurodegeneration. In a rabbit model of glaucoma, a single administration of Y-27632-loaded hollow mesoporous cerium oxide nanoparticles effectively restored corneal integrity, normalized outer nuclear layer thickness, and promoted retinal ganglion cell survival, with approximately 86% of damaged cells being rescued (Luo et al., 2021).

In addition to retinal neurodegeneration, the dysfunction of conventional aqueous humor outflow pathway is central to glaucoma pathophysiology. The trabecular meshwork (TM) is the principal site of outflow resistance, whereas chronic oxidative stress can induce loss of its cellular structure, cytoskeletal remodeling and excessive extracellular matrix deposition. These changes increase outflow resistance and thereby promote IOP elevation (Wang and Zheng, 2019). Nanozyme therapy can act at two anatomically distinct levels. On the one hand, it retains the function of TM cells to improve the outflow of aqueous humor. On the other hand, it directly protects RGCs from pressure-related oxidation and inflammatory damage.

Recent ocular-specific evidence supports the feasibility of targeting TM with a topical nanozyme formulation. Zhang et al. developed a nicotinamide-loaded cerium metal-organic framework nanozyme (NAM@CeMOF-2) for glaucoma. After topical administration, fluorescently labeled CeMOF-2 crossed the cornea and accumulated in the TM/Schlemm's canal region, with increased cerium detected in aqueous humor outflow tissue. In H_2_O_2_-stressed human TM cells, restoring NAD^+^ metabolism attenuated oxidative stress, cellular senescence and extracellular matrix accumulation. In Cav1^−^/^−^ ocular hypertensive mice, repeated NAM@CeMOF-2 eye drops reduced IOP, with a maximum reduction reported after 7 days of treatment (Zhang et al., 2025b). These findings provide direct evidence that a topical nanozyme carrier can reach the outflow pathway and protect TM-associated functions. Nevertheless, fluorescence accumulation within the TM/Schlemm's canal region should not be interpreted as evidence of uniform circumferential coverage of the entire TM. Tear loss, corneal penetration, aqueous humor convection and regional differences in outflow may produce heterogeneous exposure. Future studies should therefore quantify angular/circumferential TM coverage, local concentration-time profiles and cell type-specific uptake rather than reporting anterior segment fluorescence alone.

The second therapeutic objective is to preserve RGCs, as reducing IOP alone cannot completely prevent disease progression in all patients. Nanozymes can potentially protect RGCs by reducing mitochondrial ROS, suppressing microglial inflammatory signaling and limiting regulated cell death pathways. For instance, FeDSNP-Pa nanoassemblies were reported to catalytically eliminate excess ROS while inhibiting TNF-α/NF-κB signaling and NLRP3/caspase-1/GSDMD pyroptosis pathway, resulting in significant protection of RGCs in an elevated IOP glaucoma model (Wu et al., 2025). These results support a dual-compartment concept for glaucoma nanotherapy. Anterior-segment delivery should restore TM homeostasis and aqueous outflow to lower IOP, whereas posterior-segment delivery or sufficiently deep ocular penetration is required for direct RGC neuroprotection.

## 5 Molecular pathways of antioxidant and anti-inflammatory effects of ophthalmic nanozymes

The therapeutic effects of ophthalmic nanozymes are not limited to directly eliminating ROS but also involve modulation of redox-sensitive inflammatory and cytoprotective signaling pathways (Table 3). Among these pathways, NF-κB, Nrf2-associated antioxidant responses and NLRP3 inflammasome constitute major molecular links between oxidative stress and ocular inflammation. Excessive ROS can activate NF-κB signaling, thereby promoting the transcription of pro-inflammatory mediators such as IL-1β, IL-6, TNF-α, COX-2 and iNOS (Liu et al., 2017). By reducing the upstream ROS burden, antioxidant nanozymes can attenuate NF-κB activation and subsequently suppress the production of inflammatory cytokines. In dry eye disease, Fe/Mn dual-atom nanozyme treatment reduced NF-κB p65 and phosphorylated NF-κB p65 expression together with downstream inflammatory mediators, demonstrating that catalytic ROS removal can indirectly regulate redox-sensitive inflammatory signals.

**Table 3:** Main molecular pathways regulated by nanozymes in ocular oxidative stress and inflammation.

Pathway	Pathological role in ocular tissues	Effect of nanozymes	Downstream changes	Representative ocular evidence
Nrf2/antioxidant response	Maintains endogenous antioxidant defense under oxidative stress	Enhances Nrf2-associated antioxidant capacity and/or restores antioxidant proteins	HO-1, SOD, CAT, GPX1 (↑); ROS (↓)	Nanoceria in retinal degeneration; Fe/Mn dual-atom nanozyme in DED
NF-κB	ROS-dependent activation promotes inflammatory transcription	Attenuates ROS-dependent NF-κB signaling	p-NF-κB p65, IL-6 and TNF-α (↓)	Fe/Mn dual-atom nanozyme in DED
NLRP3 inflammasome	ROS induces NLRP3/ASC/caspase-1 assembly and maturation of inflammatory cytokines	Suppresses ROS-induced inflammasome activation	NLRP3, ASC, caspase-1, IL-1β, IL-18 (↓)	Fe/Mn dual-atom nanozyme in DED
HIF-1α/VEGF	Hypoxia/oxidative stress promotes pathological angiogenesis	ROS/H_2_O_2_ regulation and oxygen generation alleviate hypoxia signals	HIF-1α and VEGF (↓); Reduction of neovascularization	Fe-SANzyme, Cyano@Au@Ir NPs and related retinal nanozymes

The NLRP3 inflammasome represents another important downstream target of oxidative stress (Griswold et al., 2024; Niu et al., 2015). The accumulation of ROS promotes assembly of NLRP3 inflammasome and activation of ASC and caspase-1, resulting in maturation of IL-1β and IL-18 and amplification of ocular surface inflammation. Fe/Mn dual-atom nanozymes suppressed NLRP3, ASC and caspase-1 expression and reduced IL-1β and IL-18 levels in hyperosmotic corneal epithelial cells, indicating disruption of the ROS/NLRP3 inflammatory axis. Additionally, nanozymes should enhance endogenous antioxidant defense rather than merely act as direct ROS scavengers. Retinal studies using cerium oxide nanoparticles have demonstrated that the expression of Nrf2 increases after nanozyme treatment, while the expressions of HO-1, SOD1, CAT and GPX1 recover. These observations suggest that direct catalytic ROS removal and endogenous antioxidant signaling can act cooperatively to restore ocular redox homeostasis.

More importantly, these pathways are interconnected rather than independent. Activation of antioxidant defenses can decrease ROS-dependent NF-κB and NLRP3 signaling, whereas inhibition of inflammatory cascades further limits secondary ROS generation from activated immune cells. Therefore, the therapeutic effect of ophthalmic nanozymes should be regarded as a coordinated regulation of ROS and antioxidant defense network, rather than a simple chemical clearance of ROS.

## 6 Summary and future directions

This review summarizes the core characteristics and catalytic mechanisms of four types of nanozymes, including metal-based, metal oxide-based, carbon-based and composite nanozymes. By preferentially exploiting antioxidant enzyme-like activities, particularly SOD- and CAT-like activities, appropriately engineered nanozymes can sequentially remove O_2_^•-^ and H_2_O_2_, thereby alleviating pathological oxidative stress. In contrast, potential pro-oxidant POD- and OXD-like activities should be carefully controlled to avoid secondary ROS generation in ocular tissues. This catalytic selectivity, along with anti-inflammatory and anti-angiogenic effects, supports multidimensional regulation of the pathological ocular microenvironment. To overcome the limitations imposed by ocular physiological barriers such as the blood-retinal barrier and tight junctions of the corneal epithelium, modification strategies including mucin-binding-based mucosal retention, cell membrane biomimetic coating and antibody/peptide-mediated molecular targeting have significantly enhanced the enrichment efficiency of nanozymes at lesion sites. Meanwhile, the development of ROS-responsive, pH-responsive and enzyme-responsive drug release systems has further enabled precise activation and controlled release of nanozymes while reducing off-target toxicity. In terms of specific disease applications, nanozymes have achieved breakthrough progress in interventions for oxidative stress in DR, neuroprotection and neovascularization inhibition in AMD and regulation of the ROS/NLRP3 signaling axis in DED, providing novel avenues for disease-modifying therapy.

Despite the considerable clinical application potential of nanozymes in precision intervention for ophthalmic diseases, the translational pathway from laboratory research to clinical practice still faces numerous challenges. The biosafety of nanozymes requires systematic evaluation. Due to their small size and predominantly metal-based composition, they may persist in the body for extended periods, potentially posing safety concerns. Therefore, future biosafety evaluations should also integrate ocular pharmacokinetic measurements, including intraocular residence time, tissue distribution, degradation or persistence, and route-specific clearance, rather than relying solely on short-term histological toxicity analysis.

Catalytic reproducibility is a central quality control issue because the activity of nanozyme is highly sensitive to particle size, morphology, crystallinity, defect density, oxidation state, exposed active sites and surface ligand density. Even minor differences in precursor ratios, reaction temperature, pH, reaction time or purification can generate substantial batch-to-batch changes in SOD-, CAT-, POD- or OXD-like activity. For ophthalmic products, physicochemical characterization should be coupled with standardized catalytic kinetic analysis under ocular-relevant pH, ionic strength and substrate conditions. Before scaling up, critical material attributes should be linked to predefined catalytic acceptance range.

The biological environment may further change catalytic performance through the formation of protein corona. Adsorption of proteins from tears, vitreous, serum-derived components or other ocular fluids can alter the effective hydrodynamic size and surface charge of nanozymes, and can partially shield catalytic sites. More specifically, an adsorbed protein layer increases the effective particle size measured in biological fluids and can either stabilize dispersion or promote aggregation depending on protein composition and surface chemistry. Corona adsorption also shifts the apparent zeta potential toward that of the adsorbed biomolecules, thereby changing diffusion, cellular recognition and clearance. In terms of catalysis, proteins can sterically shield active sites and reduce the permeability of substrates such as H_2_O_2_ or chromogenic/electron donor molecules to the catalytic interface. Consequently, the apparent reaction rate, substrate affinity and maximum catalytic capacity measured in the medium containing proteins are quite different from those obtained in the buffer solution. For ophthalmic nanozymes, DLS/zeta-potential measurements and kinetic assays should be repeated after incubation in tears, aqueous humor or vitreous-mimicking fluids, rather than extrapolated from measurements in simple buffers.

Storage stability and sterilization are equally important, as ophthalmic formulations must remain sterile without losing their catalytic identity. During storage, nanoparticle aggregation, changes in metal oxidation state, ligand desorption and degradation of polymeric or biomimetic coatings may alter both colloidal behavior and catalytic activity. Sterilization can introduce additional changes, including that autoclaving may induce aggregation or degradation of heat-sensitive components, irradiation can modify surface chemistry or generate radicals, and sterile filtration may cause particle loss or filter blockage when particle dimensions approach the membrane pore size. Reviews of ophthalmic nanopharmaceutical manufacturing emphasize that no single sterilization method is universally suitable, and each formulation requires case-specific validation of particle size, chemical integrity, drug retention and functional performance before and after sterilization.

Large-scale manufacturing must ultimately reproduce the structural and catalytic characteristics established at laboratory scale. Multicomponent systems containing targeting ligands, responsive polymers, biological membranes or several catalytic centers are particularly vulnerable to cumulative process variability. Therefore, scalable synthesis should be developed together with standardized purification, in-process monitoring, size distribution, surface properties, sterility, ocular tolerability and catalytic activity. From a translational perspective, simplifying nanozyme architecture while preserving the minimum catalytic selectivity and delivery functions required for efficacy may provide a more realistic route than continuously increasing structural complexity to achieve current production that complies with relevant regulations.

The pathological microenvironment of retinopathy is highly complex, and single stimulus-responsive mechanisms are increasingly insufficient to meet the demands of precision clinical therapy. By developing nanosystems with multiple triggering mechanisms, further fine-tuning the stimulus-response thresholds of materials, and integrating the unique physiological characteristics of ocular barriers, the targeting efficiency of nanozyme-based drug delivery systems within complex pathological environments can be enhanced.

There are still many challenges in developing more intelligent nanozyme-based drug delivery systems for the treatment of ROS-related ophthalmic diseases in the future. The ideal drug delivery system should possess the following key features: a simple and non-invasive drug delivery method to enhance patient compliance and reduce operational risks; it has an efficient drug delivery capability, ensuring that the nanozyme can cross the eye tissue barrier and accumulate at the lesion site; it has excellent biocompatibility, especially showing no obvious toxicity or immune rejection to the eyes; it can achieve continuous and stable drug release in ocular tissues to maintain the persistence of therapeutic concentrations. In addition, it should also have good potential for clinical transformation, including large-scale production, stable storage, and compliance with regulatory requirements.

## CRediT authorship contribution statement

**Zhipeng Li:** Writing – review & editing, Funding acquisition. **Keda Yan:** Writing – original draft. **Ning Rong:** Investigation. **Shujie Sun:** Supervision. **Xuemin Liu:** Project administration. **Yu Xiu:** Methodology. **Qiusheng Zheng:** Project administration. **Yong Sun:** Writing – review & editing. **Jingliang Wu:** Writing – review & editing.

## Declaration

All the pictures obtained from the references have been permitted by the corresponding publishers.

## Declaration of competing interest

There are no conflicts to declare.

## Data Availability

No data was used for the research described in the article.
